# Analysis of clinical parameters of different types of α-thalassemia children in Hainan region, China

**DOI:** 10.7717/peerj.20586

**Published:** 2026-01-08

**Authors:** Ge Gao, Zhengnan Sun, Junhong Chen, Jinyu Kang, Fei Sun, Qi Li, Limei Fu, Yi Gong, Linna Ma, Qiuling Jie, Yanlin Ma

**Affiliations:** 1 Key Laboratory of Reproductive Health Diseases Research and Translation of Ministry of Education & Key Laboratory of Human Reproductive Medicine and Genetic Research of Hainan Province & Hainan Provincial Clinical Research Center for Thalassemia, The First Affiliated Hospital of Hainan Medical University, Hainan Medical University, Haikou, Hainan, China; 2 Hainan Academy of Medical Science, Hainan Medical University, Haikou, Hainan, China; 3 Hainan Modern Women and Children’s Hospital, Haikou, Hainan, China; 4 Henan Provincial Key Laboratory of Genetic Diseases and Functional Genomics, Medical Genetic Institute of Henan Province, Provincial People’s Hospital, Henan Provincial People’s Clinical Medical School of Zhengzhou University, Zhengzhou, Henan, China

**Keywords:** α-Thalassemia, Hb H disease, Clinical parameters, Anemia, Growth and development

## Abstract

**Background:**

Thalassemia, a hereditary hemoglobinopathy characterized by impaired hemoglobin production, results in the premature destruction of erythrocytes and consequent anemia. However, the distinct hematological parameters and phenotypic expressions associated with different α-thalassemia genotypes in the pediatric population remain inadequately characterized. Therefore, this study was designed to perform a comparative analysis of clinical parameters between pediatric patients with α-thalassemia and healthy controls, to elucidate genotype-specific disease manifestations, and to inform optimized management strategies.

**Methods:**

This retrospective cross-sectional study enrolled 160 children with genetically confirmed α-thalassemia and 105 healthy controls in Hainan. Participants were categorized into silent carrier, mild, Hb H disease, and control groups. Comprehensive assessments included hematological parameters, biochemical profiles, coagulation function, growth *Z*-scores, and serum ferritin. Group comparisons were performed across genotypes and age strata (1–5, 6–11, 12–18 years) using appropriate statistical methods.

**Results:**

Children diagnosed with Hb H disease exhibited the most severe hematological impairments, including growth retardation, elevated bilirubin levels, increased ferritin concentrations, and altered coagulation parameters. Among the genotypes studied, non-deletional types (--^SEA^/α^QS^α, --^SEA^/α^CS^α) demonstrated the most pronounced deficits. Growth *Z*-scores, encompassing weight-for-age (WAZ), height-for-age (HAZ), and body mass index-for-age (BAZ), were significantly reduced in the Hb H disease cohort, with deterioration observed as age increased. Notably, even silent carriers of the disease exhibited developmental delays during later childhood. Furthermore, iron overload and subclinical organ involvement were evident in older children affected by Hb H disease.

**Conclusions:**

The clinical phenotype of pediatric α-thalassemia is significantly influenced by both genotype and age, with non-deletional Hb H disease presenting the highest risk for systemic complications. These findings emphasize the necessity for genotype-specific monitoring, early nutritional and iron-chelation interventions, and a multidisciplinary follow-up approach to enhance long-term outcomes.

## Introduction

Thalassemia, a inherited hemoglobinopathy in China, is characterized by impaired hemoglobin synthesis, resulting in the premature destruction of erythrocytes and consequent anemia. The disorder is primarily classified into α-thalassemia and β-thalassemia, which result from mutations in the genes encoding the α-globin and β-globin chains of hemoglobin, respectively (Kattamis, Kwiatkowski & Aydinok, 2022; Piel & Weatherall, 2014). α-thalassemia has a high prevalence in southern provinces in China, especially in areas where malaria was once endemic (Kattamis, Kwiatkowski & Aydinok, 2022). Provinces like Guangdong (9.46%), Guangxi (14.13%) and Fujian have carrier rates as high as 10–15% (Lai et al., 2017), while Hainan island reports carrier rates of 17.74% (Wang et al., 2021). α-Thalassemia is primarily caused by mutations or deletions in the HBA1 and HBA2 genes, which encode the α-globin chains of hemoglobin. Non-deletional mutations often produce more severe phenotypes than deletional forms due to highly unstable hemoglobin variants. Genotype-phenotype correlations are crucial for predicting clinical outcomes (Hamid et al., 2022). These genetic alterations lead to a deficient synthesis of α-globin chains, creating an imbalance in the α- to β-globin chain ratio. This imbalance promotes the formation of unstable β-globin tetramers (Hb H) and other abnormal hemoglobin species, which ultimately leads to premature erythrocyte destruction and chronic hemolytic anemia.

The clinical severity of α-thalassemia exhibits a broad spectrum, ranging from silent carriers to severe. 1–2 gene defects lead to silent (−α/αα, α^ND^α/αα, αα^ND^/αα) or mild (--/αα, −α/−α, --α/α^ND^α, α^ND^α/α^ND^α) forms, and patients have mild anemia or no clinical symptoms (Musallam et al., 2024). The most severe form, resulting from the deletion of all four α-globin genes (Hb Bart’s hydrops fetalis), is typically fatal during the perinatal period (Horvei, MacKenzie & Kharbanda, 2021). However, a significant source of clinical complexity is hemoglobin H disease (Hb H disease), which is caused by defects in three α-globin genes (--/−α, --/α^ND^α) (Fucharoen & Viprakasit, 2009). In pediatric patients, it can lead to growth retardation, developmental delays, and a reduced quality of life. A critical complication is iron overload, which may result in irreversible organ damage (Tenuta et al., 2024). Clinical management is complicated by phenotypic heterogeneity and diagnostic complexity. Early genetic screening, individualized treatment, and lifelong monitoring of iron burden are essential.

Previous studies have predominantly focused on the molecular genetics and hematological features of α-thalassemia (Hamid et al., 2022), and most analyses have been conducted in adult populations, relatively limited attention has been paid to the broader systemic manifestations of Hb H disease in children. This gap in the literature is particularly evident for parameters such as hepatic and renal function, coagulation profiles, iron overload, and growth development. Moreover, the influence of different genotypes on these systemic parameters remains incompletely understood in pediatric populations.

To address this knowledge gap, clinical parameters were collected from 160 pediatric patients with α-thalassemia and 105 healthy controls in Hainan Island. A comprehensive analysis was conducted to compare clinical indicators across various α-thalassemia genotypes and against healthy controls, with a particular focus on Hb H disease. A detailed assessment of these clinical features is essential for developing tailored prevention and management strategies. The findings from this study are anticipated to enhance the current understanding of Hb H disease phenotypes and inform the development of optimized management strategies for affected children.

## Patients and Methods

### Recruitment and inclusion of research subjects

All participants were recruited at the First Affiliated Hospital of Hainan Medical University between May 2022 and April 2024. Genetic diagnosis for thalassemia genotypes was performed using standardized molecular techniques (BGI Genomics, Shenzhen, China) to screen for 508 known pathogenic variants in the α-globin (HBA1/HBA2) and β-globin (HBB) genes. Based on the genotyping results, 160 children with a confirmed α-thalassemia diagnosis and 105 age- and gender-matched healthy controls, who tested negative for both α- and β-thalassemia mutations, were included in the study. Participants were selected from a pediatric population (age ≤ 18 years). Exclusion criteria comprised comorbidities such as β-thalassemia, active infections, iron deficiency, other hematological or metabolic disorders, and any chronic conditions that could confound the results, including hepatic, cardiac, or renal diseases, as well as malignancies. The study protocol received approval from the Institutional Review Board (IRB) of the Ethics Committee of The First Affiliated Hospital of Hainan Medical University (Approval No.: 2022 [Scientific Research L] No. [60]). Written informed consent was obtained from all participants or their legal guardians after a comprehensive explanation of the study’s objectives and procedures. All study procedures were conducted in accordance with the principles outlined in the Declaration of Helsinki. Based on the molecular diagnosis of α-globin genotypes, participants were categorized into four groups: (1) silent carrier group (one mutated or deleted α-globin gene): −α/αα, α^ND^α/αα, αα^ND^/αα; (2) mild group (two mutated or deleted α-globin genes): --/αα, −α/−α, −α/α^ND^α, α^ND^α/α^ND^α; (3) Hb H disease group (three mutated or deleted α-globin genes): --/−α, --/α^ND^α; (4) control group (normal α-globin genotype, no mutations or deletions): αα/αα.

### Sample collection and laboratory values analysis

Peripheral venous blood samples (2–5 mL) were collected from each participant into EDTA-anticoagulated tubes. Genomic DNA was extracted using a commercial kit (QIAamp DNA Blood Mini Kit; Qiagen) according to the manufacturer’s protocol. Next-generation sequencing (NGS) was performed to screen for 508 known pathogenic variants in the HBA1 and HBA2 genes (BGI Genomics, Shenzhen, China). All participants underwent a comprehensive panel of laboratory tests, including complete blood count (CBC), biochemical analysis, coagulation profiling, and serum ferritin measurement. All data and samples were anonymized and maintained with strict confidentiality. Complete blood count (CBC) analysis was conducted using an automated hematology analyzer (Sysmex XN-3000). Coagulation profiles were assessed using an automated coagulation analyzer (Sysmex CS-2500). Biochemical parameters, including liver and renal function, were measured using an automated biochemistry analyzer (Mindray BS-2800M). Serum ferritin levels were quantified by chemiluminescence immunoassay. Samples exceeding the upper detection limit (>1,500 ng/mL) were appropriately diluted and re-analyzed to obtain accurate quantitative values.

### *Z*-score calculation

Growth status was assessed using age- and sex-specific *Z*-scores, which were calculated in accordance with the World Health Organization (WHO) Growth Standards (2006 for children aged 0–5 years and 2007 for children and adolescents aged 5–19 years). The *Z*-scores for weight-for-age (WAZ), height-for-age (HAZ), weight-for-height (WHZ), and body mass index-for-age (BAZ) were computed utilizing the WHO Anthro (version 3.2.2) and Anthro Plus (version 1.0.4) software.

### Statistical methods

All statistical analyses were performed using IBM SPSS Statistics version 25.0 (IBM Corp., Armonk, NY, USA). Continuous variables are presented as mean ± standard deviation (SD) for the clinical parameters and hemoglobin profiles of each thalassemia genotype. The normality of data distribution was assessed using the Shapiro–Wilk test. For comparisons between two groups, the independent samples *t*-test was used for normally distributed data, and the Mann–Whitney *U* test was employed for non-normally distributed data. For comparisons across more than two groups, one-way analysis of variance (ANOVA) was used for parametric data, with *post-hoc* multiple comparisons conducted using the Bonferroni correction. A two-tailed *p*-value of less than 0.05 was considered statistically significant.

## Results

### Basic information of patients

In total, 265 children were enrolled for this study, comprising 160 with α-thalassemia and 105 healthy controls. Based on their α-thalassemia genotypes, the participants were classified into four groups: silent carrier, mild, Hb H disease, and normal. The distribution of α-thalassemia genotypes across these groups is presented in Table 1. The frequencies of deletional and non-deletional mutations in the Hb H disease group were 81.63% and 18.37%, respectively. The height and weight of patients in the Hb H disease group were significantly lower than those of the normal and silent carrier groups, but not substantially different from the mild group. The proportion of Li ethnic children in the α-thalassemia groups was significantly higher than that in the normal group, increasing with the severity of α-globin gene defects. Among the α-thalassemia children, 22 (13.75%) received blood transfusions, all of whom were from the Hb H disease group. Of these, 18 patients received only occasional blood transfusions (Table 2).

**Table 1:** Distribution of α-thalassemia genotypes among different phenotype groups.

Phenotype	Genotypes	Case (*n*)	Frequency (%)
Normal	αα/αα	105	100.00
Silent carriers	−α^3.7^/αα	14	66.67
	−α^4.2^/αα	5	23.81
	α^WS^α/αα	2	9.52
	Total	21	100.00
Mild	αα/--^SEA^	30	73.17
	−α^3.7^/ − α^4.2^	3	7.32
	−α^3.7^/α^WS^α	3	7.32
	−α^4.2^/α^QS^α	2	4.88
	−α^3.7^/α^QS^α	2	4.88
	−α^3.7^/ − α^3.7^	1	2.43
	Total	41	100.00
Hb H Disease	−α^4.2^/--^SEA^	41	41.84
	−α^3.7^/--^SEA^	39	39.80
	α^WS^α/--^SEA^	10	10.20
	α^QS^α/--^SEA^	5	5.10
	α^CS^α/--^SEA^	3	3.06
	Total	98	100.00

**Table 2:** Baseline information and serum ferritin levels of different types of α-Thalassemia children in Hainan region.

**Parameter**	**Normal** **(*n* = 105)**	**Silent carrier (*n* = 21)**	**Mild** **(*n* = 41)**	**Hb H Disease** **(*n* = 98)**	***P*** **-value**
Age (years)	8.5 ± 3.4	8.52 ± 4.48	7.46 ± 3.04	7.47 ± 3.18	0.100
Gender (Male/ Female)	60/45	10/11	15/26	47/51	0.152
Height (m)	1.35 ± 0.19	1.35 ± 0.25	1.27 ± 0.19	1.22 ± 0.17^a^^b^	**<0.001**
Weight (kg)	31.63 ± 11.46	35.35 ± 15.67	28.18 ± 10.52	25.01 ± 10.83^a^^b^	**<0.001**
Nationality, *n* (%)					**<0.001**
Han	104 (99.16)	19 (92.31)	33 (81.16)	56 (58.77)	
Li	1 (0.84)	2 (7.69)	8 (17.39)	41 (40.76)	
Hui	0	0	0	1 (0.47)	
Transfusion	0	0	0	22	
Frequency of transfusions per year					
1–3 times/years	0	0	0	18	
≥4 times/years	0	0	0	4	
Nutritional anemia characteristics					
Serum Iron (µmol/L)	12.2 ± 5.05	10.87 ± 5.74	12.7 ± 4.92	13.77 ± 6.03	0.438
TIBC (µmol/L)	62.68 ± 7.98	63.97 ± 6.39	63.35 ± 5.34	54.64 ± 11.5^a^^b^^c^	**<0.001**
Transferrin (g/L)	2.53 ± 0.4	2.55 ± 0.32	2.48 ± 0.21	2.18 ± 0.38^a^^b^	**0.002**
TS(%)	19.54 ± 7.89	16.95 ± 8.99	20.3 ± 8.12	23.93 ± 11.42	0.116
Vitamin B_12_(pmol/L)	501.7 ± 196.11	575.66 ± 166.19	583.2 ± 162.3	525.32 ± 203.04	0.278

**Notes.**

Data are presented as mean ± standard deviation (SD); *P*-value stands for differences among the four groups; bold signifies *P* < 0.05.

a Compared with normal group, *P* < 0.05.

b Compared with the silent carrier group, *P* < 0.05.

c Compared with mild group, *P* < 0.05.

Abbreviations and references Serum Iron 9∼30 TIBC Total iron binding capacity 45–75 Transferrin 2.0–3.6 TS Transferrin saturation 20∼50 Vitamin B_12_ 400∼600

### Age-specific impairments in growth and development among children and adolescents with α-Thalassemia

To further investigate the effects of different types of α-thalassemia on the growth and development of children across various age groups, we categorized the enrolled children into three groups: early childhood (0–5 years), middle childhood (6–11 years), and adolescence (12–18 years). We assessed the changes in growth and development using age- and sex-specific *Z*-scores. The results indicate that as age increases, the differences in growth and development among the groups become more pronounced. In the 0–5 years group, there were no significant differences in Weight-for-Height *Z*-scores (WHZ), Height-for-Age *Z*-scores (HAZ), Weight-for-Age *Z*-scores (WAZ), and Body Mass Index *Z*-scores (BAZ) between the different α-thalassemia types and the normal group. However, with advancing age, significant differences emerged in growth and development among the groups. In the 6–11 years age group, WAZ (*P* <  0.001) and BAZ (*P* <  0.001) for the Hb H disease and mild groups were significantly lower than those of the normal control group, indicating delayed growth in weight and BMI. Even in the 12–18 years group, BAZ remained significantly lower (*P* = 0.008), suggesting that BMI development continued to be affected during adolescence. Additionally, in the 6–11 years age group, silent carriers exhibited significantly lower WAZ (*P* < 0.001) and BAZ (*P* < 0.001) compared to the normal group. By the age of 12–18 years, their HAZ also showed significant delays (*P* = 0.008). These data demonstrate age-specific effects of α-thalassemia on growth, even among silent carriers (Table 3).

**Table 3:** Age-specific differences in growth and nutritional *Z*-scores among children and adolescents with α-thalassemia subtypes.

	**Normal**	**Silent carrier**	**Mild**	**Hb H Disease**	***P*** **-value**
**1–5 years**	***n =*** **15**	***n =*** **6**	***n =*** **10**	***n =*** **29**	
WHZ	−1.23 ± 1.24	−1.01 ± 1.28	−1.07 ± 1.52	−1.15 ± 1.6	0.988
HAZ	1.15 ± 1.69	1.62 ± 2.57	0.29 ± 1.3	−0.11 ± 1.68	0.050
WAZ	−0.05 ± 0.69	0.24 ± 1.26	−0.55 ± 0.7	−0.77 ± 0.87	0.130
BAZ	−1.17 ± 1.35	−1.09 ± 1.52	−1.1 ± 1.64	−1.12 ± 1.6	0.999
**6–11 years**	***n =*** **71**	***n =*** **9**	***n =*** **28**	***n =*** **59**	
HAZ	0.98 ± 1.03	1.59 ± 0.92	0.85 ± 1.2	0.22 ± 1.81^a^^b^	**0.004**
WAZ	0.76 ± 1.02	−0.29 ± 0.53^a^	−0.2 ± 1.02^a^	−0.51 ± 0.92^a^	**<0.001**
BAZ	0.17 ± 1.47	−2.03 ± 0.74^a^	−1.44 ± 1.41^a^	−1.25 ± 2.01^a^	**<0.001**
**12–18 years**	***n =*** **19**	***n =*** **6**	***n =*** **3**	***n =*** **10**	
HAZ	−0.14 ± 1.21	−1.86 ± 1.07^a^	0.42 ± 0.33^b^	−1.05 ± 1.19	**0.008**
WAZ	0.77 ± 0.77	0.62 ± 0.33	0.27 ± 0.31	0.58 ± 0.72	0.665
BAZ	−0.36 ± 1.26	−0.42 ± 0.61	−2.77 ± 0.82^a^	−1.57 ± 1.55^a^	**0.008**

**Notes.**

Data are presented as Mean ± SD. Bold signifies *P* < 0.05.

a Compared with normal group, *P* < 0.05.

b Compared with the silent carrier group, *P* < 0.05.

Abbreviation n number WHZ weight for height *Z*-score HAZ height for age *Z*-score WAZ weight for age *Z*-score BAZ BMI for age *Z*-score

### The erythrocyte system, the platelet system varied greatly between α-thalassemia groups

To understand the anemia conditions of participants with different types of α-thalassemia, we analyzed the routine blood indicators of the enrolled participants. Compared to the normal group, the levels of erythrocyte system indicators, including hemoglobin (HGB), mean corpuscular volume (MCV), mean corpuscular hemoglobin (MCH), and mean corpuscular hemoglobin concentration (MCHC), decreased in all three α-thalassemia groups, with the most significant decrease observed in the Hb H group (*P* <  0.001), falling below clinical normal reference ranges. In contrast to the normal and silent carrier groups, the red blood cell (RBC) counts were higher in the mild and Hb H disease groups, exceeding clinical normal reference ranges. The platelet (PLT) values in the Hb H disease group were significantly higher than those in the normal group; however, this difference lacks clinical significance (Table 4). Given that many clinical hemoglobin parameters vary significantly with age and sex, we further conducted an age-stratified analysis. The results indicated that, regardless of age group, the differences in MCV, MCH, and MCHC were consistent with the overall findings. Nonetheless, the RBC, HGB, lymphocyte (LYM) count, and PLT levels of children under 11 years old aligned with the overall trend. For children over 12 years old, the HGB levels exhibited differences only in the Hb H disease group, with no significant differences observed in RBC, LYM count, and PLT levels (Table 4).

**Table 4:** Blood cells characteristics of children with α-thalassemia.

**Parameter**	**Normal**	**Silent carrier**	**Mild**	**Hb H disease**	***P*** **-value**	**References**
**Total**	***n =*** **105**	***n =*** **21**	***n =*** **41**	***n =*** **98**		
RBC (10^12^/L)	4.78 ± 0.34	5.06 ± 0.3	5.63 ± 0.34^a^^b^	5.56 ± 0.86^a^^b^	**<0.001**	4.1∼5.3
HGB (g/L)	132.51 ± 9.2	125.12 ± 12.44^a^	113.97 ± 8.9^a^^b^	98.13 ± 10.86^a^^b^^c^	**<0.001**	114∼154
HCT (%)	40.62 ± 2.7	39.16 ± 3.5	36.56 ± 2.79^a^^b^	32.14 ± 3.5^a^^b^^c^	**<0.001**	36∼47
MCV (fL)	85.23 ± 3.74	77.85 ± 7.29^a^	64.97 ± 5.19^a^^b^	57.79 ± 8.03^a^^b^^c^	**<0.001**	80∼100
MCH (pg)	27.79 ± 1.27	24.88 ± 2.6^a^	20.33 ± 1.59^a^^b^	17.65 ± 2.32^a^^b^^c^	**<0.001**	25∼34
MCHC (g/L)	326.17 ± 6.53	319.31 ± 6.58^a^	312.87 ± 5.54^a^	306.08 ± 13.83^a^^b^^c^	**<0.001**	320∼360
WBC (10^9^/L)	7.63 ± 2	7.24 ± 1.73	7.39 ± 2.52	7.41 ± 2.04	0.807	4.1∼11.0
NE# (10^9^/L)	3.46 ± 1.65	3.62 ± 1.44	3.37 ± 1.68	3.79 ± 1.61	0.416	1.8∼8.3
LYM# (10^9^/L)	3.3 ± 1.16	2.84 ± 0.7	3.23 ± 1.44	2.8 ± 0.8^a^	**0.005**	1.2∼3.8
MON# (10^9^/L)	0.42 ± 0.13	0.45 ± 0.14	0.47 ± 0.15	0.53 ± 0.64	0.305	0.14∼0.74
EO# (10^9^/L)	0.41 ± 0.34	0.33 ± 0.32	0.32 ± 0.25	0.39 ± 0.53	0.615	0∼0.68
PLT (10^9^/L)	321.51 ± 69.51	331.48 ± 50.52	353.63 ± 76.13	386.71 ± 130.08^a^	**<0.001**	150∼407
**1–5 years**	***n =*** **15**	***n =*** **6**	***n =*** **10**	***n =*** **29**		
RBC(10^12^/L)	4.73 ± 0.31	5.18 ± 0.39	5.55 ± 0.33^a^	5.64 ± 0.62^a^	**<0.001**	4.1∼5.3
HGB (g/L)	126.53 ± 8.02	117.3 ± 18.9	112.6 ± 7.26^a^	95.75 ± 10.28^a^^b^^c^	**<0.001**	114∼154
HCT (%)	38.85 ± 2.23	37.03 ± 5.31	36.17 ± 2.37	31.28 ± 2.97^a^^b^^c^	**<0.001**	36∼47
MCV (fL)	82.37 ± 3.93	71.88 ± 9.89^a^	64.53 ± 5.19^a^	56.08 ± 6.77^a^^b^^c^	**<0.001**	80∼100
MCH (pg)	26.81 ± 1.45	22.78 ± 3.51^a^	20.33 ± 1.47^a^	17.16 ± 2.09^a^^b^^c^	**<0.001**	25∼34
MCHC (g/L)	325.53 ± 7.82	316.05 ± 6.87	315.4 ± 6.04	306.32 ± 14.45	**<0.001**	320∼360
WBC (10^9^/L)	8.49 ± 2.49	6.92 ± 1.88	8.36 ± 3.38	8.33 ± 2.49	0.629	4.1∼11.0
NE# (10^9^/L)	3.08 ± 1.65	2.88 ± 1.53	3.36 ± 1.05	4.38 ± 2.13	0.074	1.8∼8.3
LYM# (10^9^/L)	4.55 ± 2.08	3.35 ± 0.83	4.19 ± 2.4	3.07 ± 1.04^a^	**0.030**	1.2∼3.8
MON# (10^9^/L)	0.43 ± 0.15	0.43 ± 0.1	0.5 ± 0.14	0.75 ± 1.13	0.560	0.14∼0.74
EO# (10^9^/L)	0.41 ± 0.32	0.23 ± 0.12	0.37 ± 0.23	0.33 ± 0.29	0.567	0∼0.68
PLT (10^9^/L)	352.27 ± 69.96	360.42 ± 41.68	403.6 ± 86.49	430.81 ± 134.27	0.118	150∼407
**6–11 years**	***n =*** **71**	***n =*** **9**	***n =*** **28**	***n =*** **59**		
RBC (10^12^/L)	4.76 ± 0.33	4.93 ± 0.28	5.68 ± 0.34^a^^b^	5.65 ± 0.92^a^^b^	**<0.001**	4.1∼5.3
HGB (g/L)	132.13 ± 7.63	126.44 ± 6.82	113.56 ± 9.38^a^^b^	99.52 ± 11.18^a^^b^^c^	**<0.001**	114∼154
HCT (%)	40.45 ± 2.19	39.39 ± 1.84	36.41 ± 2.9^a^^b^	32.5 ± 3.55^a^^b^^c^	**<0.001**	36∼47
MCV (fL)	85.12 ± 3.16	80.24 ± 3.83^a^	64.33 ± 4.69^a^^b^	56.83 ± 6.43^a^^b^^c^	**<0.001**	80∼100
MCH (pg)	27.8 ± 1.07	25.76 ± 1.4^a^	20.09 ± 1.48^a^^b^	17.43 ± 1.85^a^^b^^c^	**<0.001**	25∼34
MCHC (g/L)	326.62 ± 6.51	321.22 ± 6.04	312.15 ± 5.48^a^^b^	306.96 ± 10.89^a^^b^^c^	**<0.001**	320∼360
WBC (10^9^/L)	7.56 ± 2.03	7.66 ± 2.09	7.27 ± 2.13	7.02 ± 1.65	0.422	4.1∼11.0
NE# (10^9^/L)	3.52 ± 1.8	4.12 ± 1.45	3.44 ± 1.92	3.48 ± 1.31	0.727	1.8∼8.3
LYM# (10^9^/L)	3.15 ± 0.82	2.58 ± 0.66	3.01 ± 0.75	2.74 ± 0.57^a^	**0.005**	1.2∼3.8
MON# (10^9^/L)	0.42 ± 0.13	0.53 ± 0.17	0.48 ± 0.15	0.44 ± 0.13	**0.043**	0.14∼0.74
EO# (10^9^/L)	0.45 ± 0.37	0.43 ± 0.44	0.32 ± 0.26	0.43 ± 0.64	0.659	0∼0.68
PLT (109/L)	325.13 ± 68.87	316.11 ± 29.99	343.13 ± 65.71	389.36 ± 116.76^a^	**<0.001**	150∼407
**12–18 years**	***n =*** **19**	***n =*** **6**	***n =*** **3**	***n =*** **10**		
RBC(10^12^/L)	4.85 ± 0.4	5.12 ± 0.19	5.47 ± 0.31	4.83 ± 0.81	0.215	4.1∼5.3
HGB (g/L)	138.68 ± 11.94	130.97 ± 8.32	122.27 ± 6.77	96.9 ± 10.29^a^^b^^c^	**<0.001**	114∼154
HCT (%)	42.64 ± 3.54	40.93 ± 2.45	39.27 ± 2.35	32.53 ± 4.46^a^^b^^c^	**<0.001**	36∼47
MCV (fL)	87.86 ± 3.98	80.22 ± 5.6	72.43 ± 5.6^a^	68.41 ± 12.07^a^^b^	**<0.001**	80∼100
MCH (pg)	28.55 ± 1.35	25.67 ± 2.03	22.57 ± 1.68^a^	20.44 ± 3.51^a^^b^	**<0.001**	25∼34
MCHC (g/L)	325 ± 5.61	319.72 ± 6.94	311.1 ± 1.15	300.2 ± 24.55^a^^b^	**<0.001**	320∼360
WBC (10^9^/L)	7.19 ± 1.23	6.95 ± 0.97	5.3 ± 1.68	7.02 ± 2.04	0.256	4.1∼11.0
NE# (10^9^/L)	3.56 ± 0.92	3.6 ± 1.23	2.7 ± 0.89	3.88 ± 1.09	0.388	1.8∼8.3
LYM# (10^9^/L)	2.86 ± 0.52	2.73 ± 0.37	2.1 ± 0.7	2.39 ± 0.97	0.156	1.2∼3.8
MON# (10^9^/L)	0.45 ± 0.13	0.35 ± 0.05	0.3 ± 0.1	0.42 ± 0.18	0.242	0.14∼0.74
EO# (10^9^/L)	0.28 ± 0.17	0.28 ± 0.18	0.2 ± 0.1	0.3 ± 0.39	0.952	0∼0.68
PLT (10^9^/L)	283.74 ± 57.64	325.58 ± 74.56	285.17 ± 53.76	243.2 ± 96.12	0.187	150∼407

**Notes.**

Data are presented as mean ± standard deviation (SD); *P*-value stands for differences among the four groups; bold signifies *P* < 0.05.

a Compared with normal group, *P* < 0.05.

b Compared with the silent carrier group, *P* < 0.05.

c Compared with mild group, *P* < 0.05.

Abbreviations n number RBC red blood cell HGB hemoglobin HCT hematocrit MCV mean corpuscular volume MCH mean hemoglobin concentration MCHC mean corpuscular hemoglobin concentration WBC white blood cell NE# neutrophil count LYM# lymphocyte count MON# monocyte count EO# eosinophil count PLT platelet

### Differences in biochemical indicators in patients with different α-thalassemia genotypes

The assessment of bilirubin metabolism indicators revealed that, compared to the normal group and other thalassemia groups, the mean values of total bilirubin (TBIL), direct bilirubin (DBIL), and indirect bilirubin (IBIL) were significantly elevated in the Hb H group (*P* < 0.05). Furthermore, the evaluation of blood lipid indicators indicated that patients with Hb H disease exhibited significantly lower levels of cholesterol (CHOL), high-density lipoprotein (HDL), and low-density lipoprotein (LDL) compared to the normal group and other thalassemia groups. Notably, triglyceride (TG) levels were only reduced in the Hb H group when contrasted with the normal group. Additionally, myocardial enzyme indicators, including creatine kinase (CK), CK-MB, and lactate dehydrogenase (LDH), as well as liver function indicators such as alkaline phosphatase (ALBP) and renal function indicators like creatinine (CREA), demonstrated significant differences in the Hb H group compared to the normal group and other thalassemia groups. However, all these differential indicators remained within the clinical reference ranges and lacked clinical significance. A further age-stratified analysis indicated that for children over 12 years old, the levels of TBIL, DBIL, and IBIL were significantly increased in the Hb H group (*P* < 0.05) and exceeded the clinical normal reference ranges. Moreover, serum ferritin levels were significantly higher in the Hb H disease group than in the other groups (*P* < 0.001), with levels gradually increasing with age (Table 5).

**Table 5:** Biochemical and ferritin characteristics of children with α-thalassemia.

**Parameter**	**Normal**	**Silent carrier**	**Mild**	**Hb H disease**	***P*** **-value**	**References**
**Total**	***n =*** **105**	***n =*** **21**	***n =*** **41**	***n =*** **98**		
**Bilirubin metabolism**						
TBIL (µmol/L)	8.69 ± 4.09	9.91 ± 4	9.73 ± 4.18	18.4 ± 18.84^a^^b^^c^	**<0.001**	≤21.0
DBIL (µmol/L)	2.55 ± 1.48	2.86 ± 1.54	2.9 ± 1.33	4.89 ± 3.01^a^^b^^c^	**<0.001**	0.4∼6.8
IBIL (µmol/L)	6.13 ± 2.8	7.01 ± 2.54	6.77 ± 2.87	12.54 ± 14.64^a^^c^	**<0.001**	1.7∼17
**Lipid profile**						
CHOL (mmol/L)	4.44 ± 0.97	4.21 ± 0.99	4.3 ± 0.74	3.59 ± 0.86^a^^b^^c^	**<0.001**	<5.18
TG (mmol/L)	1.02 ± 0.47	0.89 ± 0.32	0.96 ± 0.46	0.8 ± 0.33^a^	**0.002**	<1.70
HDL (mmol/L)	1.57 ± 0.33	1.53 ± 0.39	1.51 ± 0.29	1.31 ± 0.27^a^^b^^c^	**<0.001**	1.0∼1.6
LDL (mmol/L)	2.72 ± 0.72	2.72 ± 0.94	2.62 ± 0.57	2.13 ± 0.72^a^^b^^c^	**<0.001**	≤3.3
**Myocardial enzyme**						
CK (U/L)	121.71 ± 52.34	133.73 ± 165	112.15 ± 48.59	97.1 ± 59.63^a^	**0.038**	40∼200
CK-MB (U/L)	16.6 ± 7.83	18.62 ± 7.82	19.79 ± 6.4	19.81 ± 7.97^a^	**0.017**	<25
LDH (U/L)	231.82 ± 54.2	242.24 ± 71.48	234.4 ± 26.52	258.07 ± 70^a^	**0.013**	120∼250
**Liver functions**						
ALT (U/L)	16.31 ± 12.73	15.33 ± 5.10	14.63 ± 4.12	14.78 ± 6.45	0.621	6∼29
AST (U/L)	27.17 ± 7.4	28.03 ± 5.94	27.41 ± 5.98	32.15 ± 27.38	0.199	12∼37
ALBP (g/L)	42.74 ± 2.73	42.61 ± 2.18	43.75 ± 1.84	43.69 ± 2.61^a^	**0.018**	42∼56
**Renal functions**						
BUN (mmol/L)	4.7 ± 1.18	5.19 ± 1.33	4.37 ± 1.09	4.9 ± 3.74	0.569	2.5∼6.5
CREA (µmol/L)	39.25 ± 11.07	41.01 ± 10.08	37.37 ± 10.39	33.54 ± 9.35^a^^b^	**<0.001**	33∼75
**Serum Ferritin** (ng/ml)	61.36 ± 38.79	72.03 ± 40.6	77.91 ± 48.1	182.13 ± 202.17^a^^b^^c^	**<0.001**	11.0∼306.8
**1–5 years**	***n =*** **15**	***n =*** **6**	***n =*** **10**	***n =*** **29**		
**Bilirubin metabolism**						
TBIL(µmol/L)	7.19 ± 2.6	10.13 ± 3.07	9.68 ± 4.18	17.99 ± 18.57	0.061	≤21.0
DBIL (µmol/L)	1.83 ± 0.64	2.58 ± 1.2	2.97 ± 1.23	4.4 ± 2.0^a^	**<0.001**	0.4∼6.8
IBIL (µmol/L)	5.36 ± 2.16	7.48 ± 1.93	6.61 ± 2.87	10.4 ± 6.71^a^	**0.015**	1.7∼17
**Lipid profile**						
CHOL (mmol/L)	4.52 ± 0.52	4.82 ± 1.2	4.17 ± 0.64	3.76 ± 0.75^a^^b^	**0.002**	<5.18
TG (mmol/L)	0.77 ± 0.27	0.75 ± 0.18	1.06 ± 0.46	0.81 ± 0.43	0.242	<1.70
HDL (mmol/L)	1.55 ± 0.32	1.67 ± 0.27	1.43 ± 0.31	1.28 ± 0.2^a^^b^	**0.002**	1.0∼1.6
LDL (mmol/L)	2.89 ± 0.6	3.2 ± 1.39	2.56 ± 0.42	2.3 ± 0.67^a^^b^	**0.014**	≤3.3
**Myocardial enzyme**						
CK (U/L)	129.27 ± 44.05	119.37 ± 38.1	102 ± 26.13	95.43 ± 31.33^a^	**0.023**	40∼200
CK-MB (U/L)	20.75 ± 12.02	19.18 ± 2.98	20.19 ± 7.42	22.62 ± 9.19	0.786	<25
LDH (U/L)	293.67 ± 84.15	242.5 ± 43.88	247.45 ± 36.74	273.64 ± 43.93	0.131	120∼250
**Liver functions**						
ALT (U/L)	13.93 ± 3.71	14.67 ± 4.32	16.1 ± 2.85	14.31 ± 5.89	0.724	6∼29
AST (U/L)	34.27 ± 7.97	33.22 ± 3.61	30.19 ± 7.49	32.01 ± 6.79	0.744	12∼37
ALBP (g/L)	42.45 ± 1.68	42.38 ± 2.57	43.04 ± 2.68	43.85 ± 2.12	0.164	42∼56
**Renal functions**						
BUN (mmol/L)	5.01 ± 1.37	5.72 ± 1.24	4.38 ± 1.13	4.7 ± 0.91	0.110	2.5∼6.5
CREA (µmol/L)	30.21 ± 11.47	34.95 ± 10.92	31.41 ± 10.31	28.93 ± 8.12	0.557	33∼75
**Serum ferritin** (ng/ml)	51.79 ± 34.11	84.77 ± 49.3	93.17 ± 56.41	139.99 ± 116.89^a^	**0.022**	11.0∼306.8
**6–11 years**	***n =*** **71**	***n =*** **9**	***n =*** **28**	***n =*** **59**		
**Bilirubin metabolism and LDH**						
TBIL(µmol/L)	8.44 ± 4.01	10.58 ± 4.46	9.51 ± 4.35	14.85 ± 15.27^a^	**0.002**	≤21.0
DBIL(µmol/L)	2.48 ± 1.53	3.28 ± 1.87	2.75 ± 1.38	4.28 ± 2.0^a^^c^	**<0.001**	0.4∼6.8
IBIL (µmol/L)	5.95 ± 2.67	7.28 ± 2.65	6.71 ± 3.0	10.54 ± 14.13^a^	**0.026**	1.7∼17
**Lipid profile**						
CHOL (mmol/L)	4.49 ± 1.0	3.89 ± 0.79	4.37 ± 0.81	3.6 ± 0.88^a^^c^	**<0.001**	<5.18
TG (mmol/L)	1 ± 0.38	0.81 ± 0.25	0.96 ± 0.47	0.8 ± 0.28^a^	**0.011**	<1.70
HDL (mmol/L)	1.61 ± 0.36	1.42 ± 0.36	1.52 ± 0.29	1.36 ± 0.29^a^	**<0.001**	1.0∼1.6
LDL (mmol/L)	2.71 ± 0.7	2.62 ± 0.78	2.68 ± 0.63	2.1 ± 0.74^a^^c^	**<0.001**	≤3.3
**Myocardial enzyme**						
CK (U/L)	120.42 ± 50.46	182.51 ± 247.85	117.88 ± 56.05	103.66 ± 68.7	0.051	40∼200
CK-MB (U/L)	17.43 ± 6.37	20.83 ± 10.85	19.96 ± 6.36	19.88 ± 6.84	0.120	<25
LDH (U/L)	231.71 ± 35.84	265.06 ± 95.83	232.27 ± 19.67	245.69 ± 52.64	0.086	120∼250
**Liver functions**						
ALT (U/L)	15.63 ± 6.36	20.11 ± 14.37	14.43 ± 4.23	14.42 ± 4.97	0.070	6∼29
AST (U/L)	27.27 ± 5.29	32.67 ± 9.47	26.95 ± 5.14	33.01 ± 34.34	0.383	12∼37
ALBP (g/L)	42.56 ± 2.9	42.83 ± 2.38	43.88 ± 1.45	43.65 ± 2.51	**0.039**	42∼56
**Renal functions**						
BUN (mmol/L)	4.65 ± 1.17	4.37 ± 1.19	4.35 ± 1.08	4.48 ± 1.2	0.624	2.5∼6.5
CREA (µmol/L)	37.54 ± 6.33	40.03 ± 7.42	38.55 ± 10.01	34.38 ± 7.3	**0.019**	33∼75
**Serum ferritin**(ng/ml)	63.42 ± 36.8	64.64 ± 34.06	72.09 ± 44.35	198.42 ± 234.91^a^^b^^c^	**<0.001**	11.0∼306.8
**12–18 years**	***n =*** **19**	***n =*** **6**	***n =*** **3**	***n =*** **10**		
**Bilirubin metabolism**						
TBIL (µmol/L)	10.79 ± 4.71	8.68 ± 4.48	11.97 ± 2.57	40.53 ± 24.93^a^^b^^c^	**<0.001**	≤21.0
DBIL (µmol/L)	3.37 ± 1.45	2.5 ± 1.39	4.1 ± 0.7	9.95 ± 5.26^a^^b^^c^	**<0.001**	0.4∼6.8
IBIL (µmol/L)	7.41 ± 3.4	6.15 ± 3.08	7.83 ± 1.97	30.58 ± 21.91^a^^b^^c^	**<0.001**	1.7∼17
**Lipid profile**						
CHOL (mmol/L)	4.21 ± 1.1	4.08 ± 0.9	4.13 ± 0.15	3.05 ± 0.86^a^	**0.033**	<5.18
TG (mmol/L)	1.27 ± 0.72	1.15 ± 0.4	0.7 ± 0.26	0.77 ± 0.28	0.107	<1.70
HDL (mmol/L)	1.44 ± 0.21	1.55 ± 0.52	1.67 ± 0.25	1.1 ± 0.28^a^^b^^c^	**0.006**	1.0∼1.6
LDL (mmol/L)	2.6 ± 0.86	2.38 ± 0.44	2.27 ± 0.4	1.81 ± 0.65	0.072	≤3.3
**Myocardial enzyme**						
CK (U/L)	120.56 ± 66.05	74.93 ± 21.82	92.53 ± 13.46	63.2 ± 57.34	0.072	40∼200
CK-MB (U/L)	10.22 ± 4.94	14.73 ± 4.52	16.87 ± 3.49	11.2 ± 3.58	**0.045**	<25
LDH (U/L)	183.4 ± 28.41	207.75 ± 38.15	210.83 ± 31.95	285.95 ± 162.17^a^	**0.043**	120∼250
**Liver functions**						
ALT (U/L)	20.74 ± 27.21	17 ± 6.99	11.67 ± 6.03	18.2 ± 12.91	0.906	6∼29
AST (U/L)	21.21 ± 8.85	21.88 ± 5.48	22.43 ± 5.17	27.5 ± 17.53	0.557	12∼37
ALBP (g/L)	43.63 ± 2.65	42.52 ± 1.76	44.9 ± 1.54	43.42 ± 4.3	0.728	42∼56
**Renal functions**						
BUN (mmol/L)	4.63 ± 1.12	5.9 ± 1.04	4.53 ± 1.56	7.93 ± 11.3	0.542	2.5∼6.5
CREA (µmol/L)	52.78 ± 13.33	48.55 ± 9.22	46.17 ± 3.85	42 ± 15.51	0.225	33∼75
**Serum ferritin**(ng/ml)	61.21 ± 49.31	70.38 ± 44.85	81.4 ± 61.28	208.19 ± 184.83	**0.008**	11.0∼306.8

**Notes.**

Data are presented as mean ± standard deviation (SD); *P*-value stands for differences among the four groups. Bold signifies *P* < 0.05.

a Compared with normal group, *P* < 0.05.

b Compared with the silent carrier group, *P* < 0.05.

c Compared with mild group, *P* < 0.05.

Abbreviations n number TBIL total bilirubin DBIL direct bilirubin IBIL indirect bilirubin LDH lactate dehydrogenase CHOL cholesterol TG triglyceride HDL high density lipoprotein LDL low density lipoprotein CK creatine kinase CK-MB Creatine Kinase Isoenzyme-MB; SerumFerritin ALT alanine aminotransferase AST aspartate aminotransferase ALBP alpha-1-acid glycoprotein BUN blood urea nitrogen CREA creatinine

### The level of PT and APTT significantly increased in the Hb H group

Next, we analyzed the coagulation indices of the enrolled participants. Our findings revealed that, compared to the normal group, the levels of prothrombin time (PT) and activated partial thromboplastin time (APTT) were significantly elevated in the Hb H group, while the PT level also increased in the mild group, indicating a prolonged clotting time. However, all these differential indicators remained within the clinical reference ranges and lacked clinical significance. Further age-stratified analysis indicated that, for children over 12 years old, the APTT levels were significantly increased in the Hb H group (*P* < 0.05) and exceeded the clinical normal reference ranges (Table 6). Additionally, we further stratified the patients by age and gender for comparison. Nonetheless, the sample size of each group was relatively small, and the data should be interpreted with caution (Tables S1–S3). In the future, we plan to increase the sample size for more robust comparative analysis.

**Table 6:** Coagulation function characteristics of children with α-thalassemia.

**Parameter**	**Normal**	**Silent carrier**	**Mild**	**Hb H disease**	***P*** **-value**	**References**
**Total**	***n =*** **105**	***n =*** **21**	***n =*** **41**	***n =*** **98**		
PT(s)	11.65 ± 0.67	11.85 ± 0.46	12.01 ± 0.61^a^	12.19 ± 0.85^a^	**<0.001**	9.8∼13.2
APTT(s)	30.25 ± 2.23	31.73 ± 2.7	31.91 ± 2.67	33.6 ± 7.27^a^	**<0.001**	22.5∼34.0
Fbg(g/L)	2.83 ± 0.6	3.07 ± 0.63	2.74 ± 0.43	2.72 ± 0.55	0.055	2.08∼3.85
PT-INR	1.03 ± 0.19	1.01 ± 0.05	1.03 ± 0.07	1.04 ± 0.08	0.722	0.85∼1.2
**1–5 years**	***n =*** **15**	***n =*** **6**	***n =*** **10**	***n =*** **29**		
PT(s)	11.8 ± 0.57	11.67 ± 0.5	12.01 ± 0.25	12.12 ± 0.74	0.253	9.8∼13.2
APTT(s)	30.41 ± 1.87	30.82 ± 2.18	32.44 ± 1.22	34.35 ± 11.56	0.459	22.5∼34.0
Fbg(g/L)	2.76 ± 0.82	2.8 ± 0.56	2.71 ± 0.24	2.84 ± 0.67	0.944	2.08∼3.85
PT-INR	1.03 ± 0.06	1 ± 0.06	1.02 ± 0.04	1.04 ± 0.07	0.450	0.85∼1.2
**6–11 years**	***n =*** **71**	***n =*** **9**	***n =*** **28**	***n =*** **59**		
PT(s)	11.6 ± 0.71	12.1 ± 0.39	12.01 ± 0.73	12.14 ± 0.83^a^	**<0.001**	9.8∼13.2
APTT(s)	30.22 ± 2.18	32.44 ± 2.69	31.71 ± 3.13	33.07 ± 4.5^a^	**<0.001**	22.5∼34.0
Fbg(g/L)	2.8 ± 0.54	3.31 ± 0.71	2.75 ± 0.5	2.64 ± 0.46^b^	**0.004**	2.08∼3.85
PT-INR	1.03 ± 0.23	1.03 ± 0.05	1.03 ± 0.08	1.04 ± 0.08	0.997	0.85∼1.2
**12–18 years**	***n =*** **19**	***n =*** **6**	***n =*** **3**	***n =*** **10**		
PT(s)	11.72 ± 0.6	11.65 ± 0.39	12.03 ± 0.23	12.71 ± 1.15^a^	**0.011**	9.8∼13.2
APTT(s)	30.23 ± 2.73	31.57 ± 3.32	32 ± 1.45	34.51 ± 4.39^a^	**0.02**	22.5∼34.0
Fbg(g/L)	2.99 ± 0.65	2.98 ± 0.49	2.77 ± 0.35	2.8 ± 0.6	0.822	2.08∼3.85
PT-INR	1.02 ± 0.05	1.0 ± 0	1.03 ± 0.06	1.09 ± 0.09^a^	**0.013**	0.85∼1.2

**Notes.**

Data are presented as mean ± standard deviation (SD); *P*-value stands for differences among the four groups; bold signifies *P* < 0.05.

a Compared with normal group, *P* < 0.05.

b Compared with the silent carrier group, *P* < 0.05.

c Compared with mild group, *P* < 0.05.

Abbreviations n number PT prothrombin time APTT activated partial thromboplastin time Fbg fibrinogen PT-INR prothrombin time - international normalized ratio

### Hematologic and biochemical characteristics among deletional and non-deletional Hb H genotypes

To investigate the clinical manifestations of both non-deletional and deletional Hb H disease, we classified patients with Hb H disease into these two categories and examined differences in blood cell counts, biochemical parameters, coagulation parameters, ferritin levels, and other relevant indicators. Our analysis revealed no significant differences in most of the indicators between non-deletional Hb H disease and deletional Hb H disease across the three age groups (Tables S4–S6). This finding is inconsistent with previous studies, which indicated that non-deletional Hb H disease patients exhibit more severe clinical manifestations than patients with deletional Hb H disease. We further divided non-deletional Hb H disease into --^SEA^/α^WS^α and --^SEA^/α^QS^α&--^SEA^/α^CS^α subgroups for analysis and found that --^SEA^/α^QS^α and --^SEA^/α^CS^α exhibits more severe manifestations compared to deletional Hb H disease and --^SEA^/α^WS^α. Patients with --^SEA^/α^QS^α and --^SEA^/α^CS^α genotypes showed significantly reduced red cell indices (HGB, HCT, MCV, MCH, MCHC) and elevated bilirubin (TBIL, IBIL, DBIL), which exhibited the most severe hematological impairment and hemolytic markers. --^SEA^/α^QS^α and --^SEA^/α^CS^α also had significantly lower RBC counts, PLT, lipid levels (CHOL, HDL), and myocardial enzymes (CK, CK-MB), alongside higher LDH, ALT, and BUN, indicating pronounced hemolysis, possible liver involvement, and altered metabolic and renal function compared to other genotypes. Although ferritin levels in all three groups did not reach iron overload levels, levels in the --^SEA^/α^QS^α and --^SEA^/α^CS^α groups were significantly higher than those in the --^SEA^/α^WS^α and deletion groups (Table 7). We did not further subdivide the various genotypes of Hb H disease due to the small number of patients in each group, which limited our ability to conduct more detailed analyses. We will continue to collect samples for future research.

**Table 7:** Characteristics of the non-deletional Hb H Disease genotypes (–^SEA^/α^WS^α, –^SEA^/α^QS^α, –^SEA^/α^CS^α).

**Parameter**	**Deletional** **Hb H (*n* = 80)**	--^**SEA**^**/α**^**WS**^α**(*n* = 10)**	--^**SEA**^**/α**^**QS**^**α (*n* = 3) &**--^**SEA**^**/α**^**CS**^**α (*n* = 5)**	*P* **-value**	**References**
**Blood cells**					
RBC(10^12^/L)	5.65 ± 0.83	5.92 ± 0.43	4.31 ± 0.43^a^^b^	**<0.001**	4.1∼5.3
HGB (g/L)	96.58 ± 8.9	116 ± 9.13^a^	91.38 ± 9.94^b^	**<0.001**	114∼154
HCT (%)	31.53 ± 3.04	36.87 ± 3.25^a^	32.34 ± 3.96^b^	**<0.001**	36∼47
MCV (fL)	55.5 ± 5.94	62.37 ± 3.24^a^	75.03 ± 7.29^a^^b^	**<0.001**	80∼100
MCH (pg)	17.04 ± 1.81	19.61 ± 0.64^a^	21.38 ± 3.32^a^	**<0.001**	25∼34
MCHC (g/L)	307.15 ± 9.47	314.8 ± 6.96	284.5 ± 30.33^a^^b^	**<0.001**	320∼360
WBC (10^9^/L)	7.55 ± 2.09	7.2 ± 1.18	6.24 ± 2.17	0.210	4.1∼11.0
NE# (10^9^/L)	3.88 ± 1.72	3.52 ± 0.77	3.23 ± 1.16	0.480	1.8∼8.3
LYM#(10^9^/L)	2.82 ± 0.75	2.95 ± 0.87	2.44 ± 1.11	0.360	1.2∼3.8
MON#(10^9^/L)	0.56 ± 0.7	0.43 ± 0.14	0.36 ± 0.14	0.612	0.14∼0.74
EO#(10^9^/L)	0.41 ± 0.58	0.31 ± 0.22	0.21 ± 0.18	0.536	0∼0.68
PLT (10^9^/L)	404.64 ± 127.15	324.9 ± 110.55	284.75 ± 123.72^a^	**0.012**	150∼407
**Bilirubin metabolism**					
TBIL(µmol/L)	15.67 ± 12.36	7.65 ± 2.36	59.11 ± 32.27^a^^b^	**<0.001**	≤21.0
DBIL(µmol/L)	4.56 ± 1.96	2.26 ± 0.6	11.53 ± 4.52^a^^b^	**<0.001**	0.4∼6.8
IBIL (µmol/L)	9.94 ± 5.6	5.37 ± 1.98	47.61 ± 32.44^a^^b^	**<0.001**	1.7∼17
**Lipid profile**					
CHOL(mmol/L)	3.62 ± 0.84	3.99 ± 0.61	2.83 ± 0.98^a^^b^	**0.012**	<5.18
TG (mmol/L)	0.81 ± 0.34	0.66 ± 0.24	0.9 ± 0.28	0.268	<1.70
HDL (mmol/L)	1.3 ± 0.23	1.62 ± 0.34^a^	1.03 ± 0.27^a^^b^	**<0.001**	1.0∼1.6
LDL (mmol/L)	2.15 ± 0.74	2.27 ± 0.5	1.71 ± 0.74	0.210	≤3.3
**Myocardial enzyme**					
CK (U/L)	93.38 ± 35.71	167.5 ± 137.26	46.25 ± 21.49^b^	**0.005**	40∼200
CK-MB (U/L)	20.1 ± 8.09	23.67 ± 5.19	12.03 ± 3.96^a^^b^	**<0.001**	<25
LDH (U/L)	246.7 ± 45.26	257.6 ± 35.15	372.31 ± 163.71^a^^b^	**<0.001**	120∼250
**Serum Ferritin**(ng/ml)	185.92 ± 211.03	54.77 ± 24.35	303.44 ± 151.08^b^	**0.030**	11.0∼306.8
**Liver functions**					
ALT (U/L)	14.11 ± 5.27	15.4 ± 6.31	20.63 ± 12.95^a^	**0.022**	6∼29
AST (U/L)	32.38 ± 29.77	28.6 ± 6	34.38 ± 17.65	0.895	12∼37
ALBP (g/L)	43.73 ± 2.36	43.36 ± 3.32	43.65 ± 4.15	0.915	42∼56
**Renal functions**					
BUN (mmol/L)	4.55 ± 1.11	4.41 ± 1.19	9.01 ± 12.54^a^^b^	**0.004**	2.5∼6.5
CREA (µmol/L)	0.13 ± 0.07	0.15 ± 0.09	0.11 ± 0.04	0.880	33∼75
**Coagulation function**					
PT(s)	12.17 ± 0.82	12.08 ± 0.9	12.53 ± 1.1	0.493	9.8∼13.2
APTT(s)	33.57 ± 7.93	32.78 ± 2.36	34.96 ± 3.6	0.818	22.5∼34.0
Fbg(g/L)	2.7 ± 0.55	3.04 ± 0.49	2.53 ± 0.43	0.101	2.08∼3.85
PT-INR	1.04 ± 0.08	1.04 ± 0.07	1.08 ± 0.09	0.521	0.85∼1.2

**Notes.**

Data are presented as mean ± standard deviation (SD); *P*-value stands for differences among the four groups; bold signifies *P* < 0.05.

a Compared with deletional Hb H group, *P* < 0.05.

b Compared with the –^SEA^/α^WS^α group, *P* < 0.05.

c Compared with –^SEA^/α^QS^α&–^SEA^/α^CS^α group, *P* < 0.05.

Abbreviations n number RBC red blood cell HGB hemoglobin HCT hematocrit MCV mean corpuscular volume MCH mean hemoglobin concentration MCHC mean corpuscular hemoglobin concentration WBC white blood cell NE count neutrophil count LYM count lymphocyte count MON count monocyte count EO count eosinophil count PLT platelet TBIL total bilirubin DBIL direct bilirubin IBIL indirect bilirubin LDH lactate dehydrogenase CHOL cholesterol TG triglyceride HDL high density lipoprotein LDL low density lipoprotein CK creatine kinase CK-MB Ceatine Kinase Isoenzyme-MB; Serum Ferritin ALT alanine aminotransferase AST aspartate aminotransferase ALBP alpha-1-acid glycoprotein BUN blood urea nitrogen CREA creatinine PT prothrombin time APTT activated partial thromboplastin time Fbg fibrinogen PT-INR prothrombin time - international normalized ratio

## Discussion

Hb H disease is characterized by a significant deficiency of α-globin, leading to the excessive formation of β-globin chains that result in the production of unstable hemoglobin, known as Hb H. This condition can present with varying degrees of anemia, ranging from mild to severe, and exhibits considerable clinical heterogeneity (Lal et al., 2011). The variability in clinical presentation complicates treatment strategies. Typically, patients experiencing severe anemia receive blood transfusions as symptomatic treatment; however, early monitoring and prevention of disease progression remain challenging. In this study, we employed a cross-sectional approach to stratify patients based on age, sex, and genotype. We systematically analyzed multiple dimensions of disease impact to investigate the clinical heterogeneity of pediatric α-thalassemia, focusing particularly on growth characteristics, hematological and biochemical profiles, and genotype-phenotype correlations across different subtypes. Our findings indicate a strong relationship between the clinical phenotype of α-thalassemia, particularly Hb H disease, and both the patient’s age and genotype. Furthermore, some non-deletional Hb H disease phenotypes exhibit greater severity than their deletional counterparts.

In our study, α-thalassemia was categorized into three groups: silent carriers, mild carriers, and Hb H disease. Analysis of the four groups with different genotypes and blood parameters revealed statistically significant differences in Hb, RBC count, MCV, MCH, and MCHC. The Hb levels in the Hb H group were notably lower than those in the other three groups and fell below the clinical reference values, indicating clinical diagnostic significance. Hb levels are essential for diagnosing anemia. Chronic anemia can lead to insufficient systemic oxygenation and adversely affect various physiological functions, including growth and development. In our study, the height and weight of children with thalassemia were significantly lower than those without the condition, with growth indicators in the Hb H group substantially lagging behind those in the silent carrier and mild groups. This result was further confirmed by the *Z*-scores for weight-for-age (WAZ), height-for-age (HAZ), and BMI-for-age (BAZ) (De Onis et al., 2007). Meanwhile, we also found that in patients with more severe types of non-deficient Hb H disease, the degree of anemia was more severe, and their height and weight also decreased accordingly, reflecting the systemic burden of chronic anemia. These findings align with previous research linking α-thalassemia, particularly Hb H disease, to growth delays due to chronic hemolytic anemia and associated metabolic disturbances (Delvecchio & Cavallo, 2010; Higgs, Engel & Stamatoyannopoulos, 2012). The severity of growth impairment correlated with the severity of the thalassemia type (Zhu et al., 2021), and this influence gradually intensifies with age. Notably, subtle growth disturbances were also observed in silent carriers during middle childhood and adolescence, suggesting that even milder genotypes may contribute to developmental challenges. Ineffective erythropoiesis likely exacerbates these effects by promoting red cell destruction and increasing bone marrow stress, which may alter cytokine profiles and hormonal regulation (Lal et al., 2024). Chronic anemia significantly affects children’s growth and development of α-thalassemia, especially hindering physical maturation and adolescent development (Harteveld et al., 2022). These findings underscore the critical need for genotype-specific monitoring and early intervention to optimize developmental outcomes in affected children. We advocate for an integrated, multidisciplinary management approach that includes routine hematologic monitoring, regular growth assessments and targeted nutritional support to improve long-term outcomes for pediatric patients.

Our study demonstrated that the TBIL and DBIL levels in the silent carrier, mild, and Hb H disease groups were all significantly higher than those in the normal group. Notably, the Hb H group exhibited a significantly greater increase compared to the silent carrier and mild Hb H disease groups. These differences are clinically significant among children over 12 years of age. Previous studies have reported similar patterns, indicating that the production of elongated or unstable α-globin chains leads to precipitation within erythroid precursors, thereby exacerbating hemolysis and bone marrow stress (Lal et al., 2024). Hemolysis can result in an increase in bilirubin decomposition, subsequently elevating bilirubin-related indicators. Concurrently, heightened hemolysis can lead to the accumulation of ferritin, which aligns with our observed results. As patients with Hb H age, their ferritin levels tend to rise, suggesting that the impact of hemolysis intensifies with age. This finding underscores the importance of early recognition and intervention for emerging issues in these patients.

Evidence suggests that β-thalassemia induces a hypercoagulable state, attributed to factors such as the exposure of phosphatidylserine on red blood cells, the increased fragility of these cells, and the absence of the spleen. Laboratory tests confirm elevated coagulation markers in thalassemia, particularly among patients who have undergone splenectomy and are not regularly transfused (Taher et al., 2008). Although research on α-thalassemia is limited, our findings indicate that PT and APTT in the three thalassemia groups were significantly higher than in the normal group. This suggests that as the severity of the disease increases, the risk of thrombosis also rises due to abnormal coagulation in patients. Notably, the difference in APTT was clinically significant only in patients aged 12 years, indicating that this effect may also be cumulative with age. Therefore, the coagulation function of patients with α-thalassemia, particularly those with Hb H disease, should be monitored concurrently before the age of 12 to detect abnormalities early and prevent thrombosis.

The values of PLT, platelet large cell ratio (PLC-R), mean platelet volume (MPV), and plateletcrit (PCT) in the α-thalassemia groups were significantly higher than those in the normal group, particularly in the Hb H disease subgroup. However, all these differences remain within the normal clinical reference range and lack significant clinical relevance. This observation may be attributed to the correlation between disease severity and age. Symptoms during childhood are generally mild, and no significant clinical abnormalities have been observed thus far, suggesting a state of clinical sub-health.

Despite the valuable insights provided by this study regarding the multisystemic effects of different α-thalassemia genotypes in children and adolescents, several limitations warrant acknowledgment. Our cross-sectional study, employing an age- and sex-stratified approach, has established a robust framework for evaluating the multifaceted impact of α-thalassemia across pediatric populations. We have elucidated genotype-specific differences, particularly the pronounced severity of non-deletional Hb H disease. However, certain limitations must be recognized. Potential gender imbalances within subgroups may have influenced sex-specific comparisons, and the absence of longitudinal data restricts our ability to fully characterize long-term disease trajectories. To address these gaps, we propose that future research prioritize larger-scale, longitudinal studies that incorporate comprehensive clinical and biochemical markers while controlling for treatment-related variables. Such studies would more effectively delineate the progression of growth impairments and iron overload from childhood into adulthood.

## Conclusion

Our analysis highlights the significant clinical and biochemical impacts of different types of α-thalassemia, particularly Hb H disease. We observed critical differences in hematological indices, iron metabolism, and biochemical indicators among patients, which varied according to the severity of their condition. These findings underscore the necessity for regular monitoring and tailored management strategies to mitigate complications and improve the quality of life for these patients. Therefore, it is essential to pay attention to the anemia, growth and development, and ferritin levels of affected children and to intervene promptly to promote their normal growth and development, especially in patients with Hb H disease. By enhancing our understanding of the clinical heterogeneity of α-thalassemia and its management, we can better address the specific needs of this patient population.

## Supplemental Information

10.7717/peerj.20586/supp-1 Supplemental Information 1 Raw data All the information of the collected data and contains four tables, each of which is for different groups of information. The baseline information represented by numbers is described below, and the rest are the numerical values of clinical testing items, whose units have been shown in detail in the table 1–7. Note: Nation, Han = 1,Hui = 2, Li = 3, Miao = 4 Gender: boy = 1, girl = 2

10.7717/peerj.20586/supp-2 Supplemental Information 2 Hematological and biochemical characteristics of children aged 1–5 years, boys and girls, with α-thalassemia

10.7717/peerj.20586/supp-3 Supplemental Information 3 Supplementary table 2. Hematological and biochemical characteristics of children aged 6–11 years (boys and girls) with α-thalassemia

10.7717/peerj.20586/supp-4 Supplemental Information 4 Hematological and biochemical characteristics of children aged 12–18 years (boys and girls) with α-thalassemia

10.7717/peerj.20586/supp-5 Supplemental Information 5 Hb H disease blood characteristics of children

10.7717/peerj.20586/supp-6 Supplemental Information 6 Biochemical and ferritin characteristics of children with deletional *vs.* non-deletional Hb H disease

10.7717/peerj.20586/supp-7 Supplemental Information 7 Coagulation function characteristics of children with deletional *vs.* non-deletional Hb H disease

10.7717/peerj.20586/supp-8 Supplemental Information 8 Hematologic and biochemical characteristics among deletional and non-deletional Hb H Disease difference genotypes

## Additional Information and Declarations

### Data Availability

The following information was supplied regarding data availability: The raw measurements are available in the Supplementary Files.
