# Supplementary material for: Analysis of clinical parameters of different types of α-thalassemia children in Hainan region, China
Source: PeerJ. 2026 Jan 8;14:e20586. doi: 10.7717/peerj.20586 (PMC12790785; doi:10.7717/peerj.20586)
Supplement: Supplemental Information 2 [file peerj-14-20586-s002.docx]

**Supplementary table1. Hematological and biochemical characteristics of children aged 1–5 years（boys and girls）with α-thalassemia**

| **Parameter** | **Normal** | | | **Silent carrier** | | **Mild** | | **Hb H Disease** | | ***P1*-value** | ***P2-*value** | **References** |  |
| --- | --- | --- | --- | --- | --- | --- | --- | --- | --- | --- | --- | --- | --- |
|  | **Boys (****n=9)** | | **Girls(n=6)** | **Boys(n=4)** | **Girls(n=2)** | **Boys(n=4)** | **Girls(n=6)** | **Boys(n=12)** | **Girls(n=17)** | **Boys** | **Girls** |  |  |
| Hemolysis |  | |  |  |  |  |  |  |  |  |  |  |  |
| RBC(10^12^/L) | 4.66±0.33 | | 4.85±0.26 | 5.13±0.49 | 5.3±0 | 5.43±0.42 | 5.63±0.27 | 5.75±0.58 **^a^** | 5.56±0.65 | **<0.001** | 0.41 | 4.1~5.3 |  |
| HGB (g/L) | 125.33±7.57 | | 128.33±9.05 | 121.45±11.94 | 109±33.94 | 111.25±3.3 **^a^** | 113.5±9.27 | 93.08±8.22 **^abc^** | 97.64±11.37**^a^** | **<0.001** | **<0.001** | 114~154 |  |
| HCT (%) | 38.29±2.06 | | 39.7±2.39 | 38.2±3.42 | 34.7±9.48 | 35.43±1.44 | 36.67±2.85 | 30.37±1.91 **^abc^** | 31.92±3.45 **^a^** | **<0.001** | **<0.001** | 36~47 |  |
| MCV (fL) | 82.86±4.87 | | 81.65±2.06 | 74.9±3.87 | 65.85±18.31 | 65.45±7.1**^a^** | 63.92±4.13 | 53.23±4.44 **^abc^** | 58.09±7.51 **^a^** | **<0.001** | **<0.001** | 80~100 |  |
| MCH (pg) | 27.07±1.46 | | 26.42±1.47 | 23.83±1.43 **^a^** | 20.7±6.51 | 20.73±2.13 **^ab^** | 20.07±0.96 | 16.27±0.82 **^abc^** | 17.79±2.49 **^a^** | **<0.001** | **<0.001** | 25~34 |  |
| MCHC (g/L) | 326.89±5.16 | | 323.5±10.97 | 317.8±34.22 | 312.5±12.02 | 316.75±3.86 | 314.5±7.37 | 316.75±3.86 **^a^** | 306.08±12.38 **^a^** | **0.009** | 0.24 | 320~360 |  |
| TBIL(μmol/L) | 7.36±2.72 | | 6.95±2.64 | 10.38±3.73 | 9.65±2.19 | 9.6±4.72 | 9.73±4.25 | 22.3±26.15 | 14.95±10.47 | 0.248 | 0.204 | ≤21.0 |  |
| DBIL(μmol/L) | 1.89±0.55 | | 1.75±0.8 | 2.83±1.47 | 2.1±0 | 2.78±1.56 | 3.1±1.11 | 4.5±1.29 **^a^** | 4.32±2.42 | **<0.001** | **0.046** | 0.4~6.8 |  |
| IBIL (μmol/L) | 5.47±2.42 | | 5.2±1.9 | 7.45±2.14 | 7.55±2.19 | 6.73±3.05 | 6.53±3.04 | 10.03±4.05 **^a^** | 10.67±8.2 | **0.03** | 0.283 | 1.7~17 |  |
| LDH (U/L) | 280.56±94.47 | | 313.33±69.09 | 227.75±17.97 | 272±77.78 | 245.75±51.09 | 248.58±29.32 | 276.42±42.05 | 271.68±46.39 | 0.458 | 0.184 | 120~250 |  |
| Leukocytes |  | |  |  |  |  |  |  |  |  |  |  |  |
| WBC (10^9^/L) | 8.11±2.89 | | 9.07±1.82 | 7.18±2.25 | 6.4±1.27 | 7.55±1.83 | 8.9±4.2 | 8.53±2.24 | 8.2±2.71 | 0.766 | 0.674 | 4.1~11.0 |  |
| NE# (10^9^/L) | 2.32±0.64 | | 4.22±2.1 | 3.23±1.85 | 2.2±0.14 | 3.1±0.45 | 3.53±1.33 | 4.05±1.44 **^a^** | 4.62±2.52 | **0.03** | 0.442 | 1.8~8.3 |  |
| LYM#(10^9^/L) | 4.86±2.57 | | 4.08±1.06 | 3.28±0.68 | 3.5±1.41 | 3.73±1.76 | 4.5±2.88 | 3.45±1.03 | 2.81±0.99 | 0.276 | 0.106 | 1.2~3.8 |  |
| MON#(10^9^/L) | 0.36±0.11 | | 0.53±0.15 | 0.43±0.13 | 0.45±0.07 | 0.48±0.15 | 0.52±0.15 | 0.66±0.27 **^a^** | 0.81±1.48 | **0.016** | 0.911 | 0.14~0.74 |  |
| EO#(10^9^/L) | 0.54±0.35 | | 2.48±1.63 | 0.25±0.13 | 3.0±0.85 | 0.38±0.36 | 4.75±3.23 | 0.39±0.24 | 3.36±2.75 | 0.36 | 0.742 | 0~0.68 |  |
| Platelets |  | |  |  |  |  |  |  |  |  |  |  |  |
| PLT (10^9^/L) | 337.44±59.17 | | 374.5±84.36 | 382.63±30.32 | 316±2.83 | 404.5±88.81 | 403±93.44 | 449.42±122.86 | 417.68±143.97 | 0.084 | 0.677 | 150~407 |  |
| Lipid profile |  | |  |  |  |  |  |  |  |  |  |  |  |
| CHOL(mmol/L) | 4.41±0.55 | | 4.68±0.48 | 4.98±1.43 | 4.5±0.85 | 3.93±0.42 | 4.33±0.74 | 3.63±0.59 | 3.84±0.85 | 0.14 | 0.122 | <5.18 |  |
| TG (mmol/L) | 0.78±0.32 | | 0.77±0.2 | 0.78±0.21 | 0.7±0.14 | 1.3±0.67 | 0.9±0.2 | 0.65±0.29 | 0.92±0.48 | 0.37 | 0.777 | <1.70 |  |
| HDL (mmol/L) | 1.6±0.33 | | 1.47±0.33 | 1.7±0.24 | 1.6±0.42 | 1.3±0.12 | 1.52±0.38 | 1.27±0.21 | 1.29±0.21 **^ab^** | **0.008** | 0.195 | 1.0~1.6 |  |
| LDL (mmol/L) | 2.73±0.67 | | 3.12±0.44 | 3.53±1.66 | 2.55±0.35 | 2.48±0.05 | 2.62±0.55 | 2.15±0.4 | 2.41±0.81 | 0.458 | 0.222 | ≤3.3 |  |
| Myocardial enzyme | | |  |  |  |  |  |  |  |  |  |  |  |
| CK (U/L) | 135.67±38.05 | | 119.67±54.14 | 119.55±43.12 | 119±41.01 | 102.05±39.64 | 101.97±16.91 | 101.42±30.94 | 91.21±31.84 | 0.182 | 0.345 | 40~200 |  |
| CK-MB (U/L) | 22.67±15.1 | | 17.87±4.81 | 18.83±3.28 | 19.9±3.25 | 19.38±8.33 | 20.73±7.52 | 25.18±9.57 | 20.81±8.74 | 0.697 | 0.878 | <25 |  |
| Ferritin |  | |  |  |  |  |  |  |  |  |  |  |  |
| Serum Ferritin  (ng/ml) | 57.37±41.82 | | 43.42±17.85 | 100.33±54.2 | 53.65±20.86 | 93.83±60.03 | 92.73±59.7 | 114.76±97.48 | 157.81±128.7 | 0.391 | 0.101 | 11.0~306.8 |  |
| Liver functions |  | |  |  |  |  |  |  |  |  |  |  |  |
| ALT (U/L) | 14.89±3.79 | | 12.5±3.39 | 15.0±5.29 | 14±2.83 | 16.75±1.89 | 15.67±3.45 | 14.17±2.73 | 14.41±7.46 | 0.636 | 0.843 | 6~29 |  |
| AST (U/L) | 34.56±9.59 | | 33.83±5.49 | 31.58±3.28 | 36.5±0.71 | 32.65±10.9 | 28.55±4.66 | 32.83±5.83 | 31.43±7.51 | 0.918 | 0.391 | 12~37 |  |
| ALBP (g/L) | 42.09±1.17 | | 42.98±2.27 | 43.5±2.45 | 40.15±0.21 | 41.48±2.53 | 44.08±2.42 | 43.73±1.36 | 43.93±2.57 | 0.055 | 0.204 | 42~56 |  |
| Renal functions |  | |  |  |  |  |  |  |  |  |  |  |  |
| BUN (mmol/L) | 5.42±1.38 | | 4.4±1.22 | 6.25±1.16 | 4.65±0.49 | 5.23±1.19 | 3.82±0.71 | 4.78±0.91 | 4.64±0.93 | 0.176 | 0.343 | 2.5~6.5 |  |
| CREA (μmol/L) | 30.34±10.56 | | 30.02±13.78 | 40.53±8.24 | 23.8±4.38 | 34.85±12.93 | 29.12±8.67 | 27.83±8.28 | 29.72±8.17 | 0.151 | 0.861 | 33~75 |  |
| Coagulation Function | | |  |  |  |  |  |  |  |  |  |  |  |
| PT(s) | | 11.69±0.62 | 11.97±0.49 | 11.8±0.42 | 11.4±0.71 | 11.95±0.1 | 12.05±0.32 | 12.28±0.68 | 12±0.78 | 0.151 | 0.668 | 9.8~13.2 |  |
| APTT(s) | | 30.62±1.65 | 30.1±2.29 | 31.58±0.61 | 29.3±3.96 | 32.1±0 | 32.67±1.58 | 37.75±17.63 | 31.95±2.32 | 0.533 | 0.122 | 22.5~34.0 |  |
| Fbg(g/L) | | 2.33±0.44 | 3.4±0.86 | 2.55±0.33 | 3.3±0.71 | 2.7±0.2 | 2.72±0.28 | 2.68±0.5 | 2.96±0.76 | 0.321 | 0.379 | 2.08~3.85 |  |
| PT-INR | | 1.01±0.06 | 1.05±0.05 | 1.03±0.05 | 53.65±20.86 | 1.03±0.05 | 92.73±59.7 | 1.05±0.07 | 157.81±128.7 | 0.546 | 0.263 | 0.85~1.2 |  |

Notes: Data are presented as mean ± standard deviation (SD);P1-value stands for Boys differences among the four groups, P2-value stands for Girls differences among the four groups, Bold Signifies *P*<0.05;**^a^** Compared with normal group, *P* <0.05; **^b^** Compared with the Silent carrier group, *P* <0.05; **^c^** Compared with mild group, *P* <0.05;

Abbreviations: RBC, red blood cell; HGB, hemoglobin:; HCT, hematocrit; MCV, mean corpuscular volume; MCH, mean hemoglobin concentration; MCHC, mean corpuscular hemoglobin concentration; TBIL, total bilirubin; DBIL, direct bilirubin; IBIL, indirect bilirubin; LDH, lactate dehydrogenase ;WBC, white blood cell; NE#, neutrophil count; LYM#, lymphocyte count; MON#, monocyte count; EO#, eosinophil count; PLT, platelet; CHOL, cholesterol; TG, triglyceride; HDL, high density lipoprotein; LDL, low density lipoprotein; CK, creatine kinase; CK-MB, Creatine Kinase Isoenzyme-MB; Serum Ferritin; ALT, alanine aminotransferase; AST, aspartate aminotransferase; ALBP, alpha-1-acid glycoprotein; BUN, blood urea nitrogen; CREA, creatinine; PT, prothrombin time; APTT, activated partial thromboplastin time; Fbg, fibrinogen; PT-INR, prothrombin time - international normalized ratio.
