# Supplementary material for: Analysis of clinical parameters of different types of α-thalassemia children in Hainan region, China
Source: PeerJ. 2026 Jan 8;14:e20586. doi: 10.7717/peerj.20586 (PMC12790785; doi:10.7717/peerj.20586)
Supplement: Supplemental Information 3 [file peerj-14-20586-s003.docx]

**Supplementary table 2. Hematological and biochemical characteristics of children aged 6-11 years (boys and girls)with α-thalassemia**

| **Parameter** | **Normal** | | **Silent carrier** | | **Mild** | | **Hb H Disease** | | ***P1*-value** | ***P2*-value** | **References** |
| --- | --- | --- | --- | --- | --- | --- | --- | --- | --- | --- | --- |
|  | **Boys(n=41)** | **Girls(n=30)** | **Boys(n=3)** | **Girls(n=6)** | **Boys(n=11)** | **Girls(n=17)** | **Boys(n=34)** | **Girls(n=25)** | **Boys** | **Girls** |  |
| Hemolysis |  |  |  |  |  |  |  |  |  |  |  |
| RBC(10^12^/L) | 4.77±0.33 | 4.76±0.33 | 5.07±0.31 | 4.87±0.27 | 5.86±0.24**^a^** | 5.56±0.35 **^a^** | 5.76±0.99**^a^** | 5.5±0.8**^a^** | **<0.001** | **<0.001** | 4.1~5.3 |
| HGB (g/L) | 132.17±7.42 | 132.07±8.03 | 123±5.2 | 128.17±7.28**^a^** | 118.09±7.4**^a^** | 110.64±9.54 **^ab^** | 100.7±10.58**^abc^** | 97.9±11.97 **^abc^** | **<0.001** | **<0.001** | 114~154 |
| HCT (%) | 40.56±2.04 | 40.3±2.4 | 38.27±1.07 | 39.95±1.96 | 37.84±2.19**^a^** | 35.48±2.98 **^ab^** | 32.87±3.38 **^abc^** | 31.99±3.78 **^abc^** | **<0.001** | **<0.001** | 36~47 |
| MCV (fL) | 85.27±3.64 | 84.92±2.42 | 75.97±3.78 **^a^** | 82.38±1.16**^a^** | 64.65±4.31 **^ab^** | 64.12±5.04 **^ab^** | 55.3±5.39 **^abc^** | 58.92±7.22 **^abc^** | **<0.001** | **<0.001** | 80~100 |
| MCH (pg) | 27.79±1.23 | 27.82±0.84 | 24.47±1.96 **^a^** | 26.4±0.31 | 20.19±1.36 **^ab^** | 20.02±1.59 **^ab^** | 17.02±1.72 **^abc^** | 17.98±1.91 **^abc^** | **<0.001** | **<0.001** | 25~34 |
| MCHC (g/L) | 325.85±6.02 | 327.67±7.09 | 321.67±9.71 | 321±4.52 | 312.36±5.41**^a^** | 312.02±5.68 **^a^** | 307.83±9.21 **^ab^** | 305.78±12.95 **^ab^** | **<0.001** | **<0.001** | 320~360 |
| TBIL(μmol/L) | 7.81±3.03 | 9.29±4.98 | 8.07±1.11 | 11.83±5.06 | 8.13±3.37 | 10.41±4.76 | 11.77±6.06**^a^** | 19.03±21.93**^a^** | **0.002** | **0.046** | ≤21.0 |
| DBIL(μmol/L) | 2.28±1.06 | 2.76±1.99 | 2.33±0.81 | 3.75±2.13 | 2.27±0.95 | 3.06±1.54 | 3.81±1.89**^ac^** | 4.92±2.02**^ac^** | **<0.001** | **<0.001** | 0.4~6.8 |
| IBIL (μmol/L) | 5.53±2.17 | 6.53±3.18 | 5.73±0.4 | 8.05±3.01 | 5.82±2.46 | 7.28±3.25 | 7.95±4.5**^a^** | 14.07±20.78 | **0.017** | 0.123 | 1.7~17 |
| LDH (U/L) | 233.33±37.2 | 229.5±34.38 | 217.33±28.01 | 288.92±111.04 | 231±21.91 | 233.09±18.74 | 239.56±35.11 | 254.02±69.83 | 0.660 | 0.060 | 120~250 |
| Leukocytes |  |  |  |  |  |  |  |  |  |  |  |
| WBC (10^9^/L) | 7.55±1.9 | 7.57±2.23 | 7.8±3.75 | 7.58±1.14 | 6.5±1.37 | 7.76±2.4 | 7.32±1.77 | 6.61±1.4 | 0.404 | 0.211 | 4.1~11.0 |
| NE# (10^9^/L) | 3.42±1.47 | 3.65±2.19 | 3.73±2.14 | 4.32±1.19 | 2.75±0.77 | 3.89±2.3 | 3.72±1.49 | 3.15±0.94 | 0.27 | 0.422 | 1.8~8.3 |
| LYM#(10^9^/L) | 3.14±0.84 | 3.17±0.79 | 2.77±0.96 | 2.48±0.55 | 2.95±0.52 | 3.05±0.88 | 2.72±0.61 | 2.77±0.52 | 0.096 | 0.074 | 1.2~3.8 |
| MON#(10^9^/L) | 0.43±0.14 | 0.4±0.11 | 0.43±0.06 | 0.58±0.18 | 0.48±0.14 | 0.48±0.16 | 0.46±0.14 | 0.42±0.13**^b^** | 0.617 | **0.014** | 0.14~0.74 |
| EO#(10^9^/L) | 0.53±0.41 | 0.34±0.26 | 0.83±0.59 | 0.23±0.19 | 0.3±0.17 | 0.33±0.31 | 0.51±0.78 | 0.32±0.37 | 0.479 | 0.791 | 0~0.68 |
| Platelets |  |  |  |  |  |  |  |  |  |  |  |
| PLT (10^9^/L) | 318.63±65.24 | 334±73.73 | 329.33±30.92 | 309.5±29.99 | 361.27±57.84 | 331.38±69.45 | 407.04±116.85**^a^** | 365.32±114.58 | **<0.001** | 0.373 | 150~407 |
| Lipid profile | |  |  |  |  |  |  |  |  |  |  |
| CHOL(mmol/L) | 4.56±1.02 | 4.39±0.99 | 4.47±1 | 3.6±0.55 | 4.44±0.68 | 4.32±0.91 | 3.64±0.7**^a^** | 3.55±1.1**^ac^** | **<0.001** | **0.009** | <5.18 |
| TG (mmol/L) | 1±0.39 | 1.01±0.37 | 0.83±0.25 | 0.8±0.27 | 0.95±0.44 | 0.96±0.5 | 0.77±0.26 | 0.84±0.3 | 0.051 | 0.309 | <1.70 |
| HDL (mmol/L) | 1.64±0.4 | 1.58±0.3 | 1.27±0.29 | 1.5±0.39 | 1.58±0.25 | 1.48±0.31 | 1.45±0.28 | 1.25±0.26**^a^** | **0.048** | **0.001** | 1.0~1.6 |
| LDL (mmol/L) | 2.71±0.65 | 2.7±0.78 | 3.3±0.56 | 2.28±0.67 | 2.7±0.69 | 2.66±0.61 | 2.04±0.55**^a^** | 2.18±0.95 | **<0.001** | 0.080 | ≤3.3 |
| Myocardial enzyme | |  |  |  |  |  |  |  |  |  |  |
| CK (U/L) | 123.84±55.12 | 115.73±43.76 | 95.67±23.69 | 225.93±302.12 | 149.38±67.66 | 97.49±36.29 | 114.92±83.31 | 88.34±37.91 | 0.463 | **0.009** | 40~200 |
| CK-MB (U/L) | 16.73±6.55 | 18.4±6.09 | 17.6±3.62 | 22.45±13.17 | 24.33±5.23**^a^** | 17.14±5.44 | 19.67±6.23 | 20.17±7.72 | **0.005** | 0.345 | <25 |
| Ferritin |  |  |  |  |  |  |  |  |  |  |  |
| SerumFerritin  (ng/ml) | 64.32±38.47 | 62.2±35 | 75.77±50.91 | 59.08±26.61 | 54.87±46.27 | 83.23±40.56 | 143.36±175.56**^a^** | 273.31±284.32**^abc^** | **0.019** | **<0.001** | 11.0~306.8 |
| Liver functions | |  |  |  |  |  |  |  |  |  |  |
| ALT (U/L) | 16.17±6.32 | 14.9±6.45 | 24.67±22.81 | 17.83±10.19 | 14.55±3.33 | 14.35±4.82 | 13.91±5.07 | 15.12±4.85 | 0.41 | 0.674 | 6~29 |
| AST (U/L) | 27.37±5.99 | 27.13±4.25 | 34.33±9.24 | 31.83±10.34 | 27.3±5.93 | 26.72±4.74 | 28.58±5.32 | 39.05±52.39 | 0.22 | 0.460 | 12~37 |
| ALBP (g/L) | 42.89±2.77 | 42.1±3.07 | 44.2±0.6 | 42.15±2.69 | 43.33±1.22 | 44.24±1.51 | 43.59±2.78 | 43.74±2.14 | 0.625 | **0.016** | 42~56 |
| Renal functions | |  |  |  |  |  |  |  |  |  |  |
| BUN (mmol/L) | 4.81±1.16 | 4.45±1.16 | 4.47±0.75 | 4.32±1.42 | 4.39±1.05 | 4.32±1.13 | 4.6±1.25 | 4.32±1.14 | 0.706 | 0.976 | 2.5~6.5 |
| CREA (μmol/L) | 37.58±6.06 | 37.48±6.78 | 41.03±2.17 | 39.53±9.23 | 41.32±12.12 | 36.76±8.29 | 33.76±7.09 | 35.22±7.63 | 0.013 | 0.553 | 33~75 |
| Coagulation Function | |  |  |  |  |  |  |  |  |  |  |
| PT(s) | 11.55±0.79 | 11.66±0.58 | 12.47±0.42 | 11.92±0.22 | 12.19±1.03 | 11.89±0.44 | 11.98±0.66 | 12.36±0.99**^a^** | **0.016** | **0.005** | 9.8~13.2 |
| APTT(s) | 29.94±1.85 | 30.6±2.56 | 34.23±4.14 | 31.55±1.35 | 32.21±4.72 | 31.39±1.52 | 33.16±3.09**^a^** | 32.96±5.97 | **<0.001** | 0.167 | 22.5~34.0 |
| Fbg(g/L) | 2.8±0.58 | 2.8±0.49 | 3.37±0.98 | 3.28±0.65 | 2.55±0.51 | 2.87±0.46 | 2.71±0.52 | 2.56±0.36**^b^** | 0.144 | **0.006** | 2.08~3.85 |
| PT-INR | 1±0.08 | 1.07±0.33 | 1.07±0.06 | 1.02±0.04 | 1.05±0.1 | 1.02±0.05 | 1.03±0.07 | 1.06±0.09 | 0.197 | 0.828 | 0.85~1.2 |

Notes: Data are presented as mean ± standard deviation (SD);P1-value stands for Boys differences among the four groups, P2-value stands for Girls differences among the four groups,;Bold Signifies *P*<0.05;**^a^** Compared with normal group, *P* <0.05; **^b^** Compared with the Silent carrier group, *P* <0.05; **^c^** Compared with mild group, *P* <0.05;

Abbreviations: RBC, red blood cell; HGB, hemoglobin:; HCT, hematocrit; MCV, mean corpuscular volume; MCH, mean hemoglobin concentration; MCHC, mean corpuscular hemoglobin concentration; TBIL, total bilirubin; DBIL, direct bilirubin; IBIL, indirect bilirubin; LDH, lactate dehydrogenase; WBC, white blood cell; NE#, neutrophil count; LYM#, lymphocyte count; MON#, monocyte count; EO#, eosinophil count; PLT, platelet; CHOL, cholesterol; TG, triglyceride; HDL, high density lipoprotein; LDL, low density lipoprotein; CK, creatine kinase; CK-MB, Creatine Kinase Isoenzyme-MB; Serum Ferritin; ALT, alanine aminotransferase; AST, aspartate aminotransferase; ALBP, alpha-1-acid glycoprotein; BUN, blood urea nitrogen; CREA, creatinine; PT, prothrombin time; APTT, activated partial thromboplastin time; Fbg, fibrinogen; PT-INR, prothrombin time - international normalized ratio.
