# Supplementary material for: Analysis of clinical parameters of different types of α-thalassemia children in Hainan region, China
Source: PeerJ. 2026 Jan 8;14:e20586. doi: 10.7717/peerj.20586 (PMC12790785; doi:10.7717/peerj.20586)
Supplement: Supplemental Information 4 [file peerj-14-20586-s004.docx]

**Supplementary table 3.Hematological and biochemical characteristics of children aged 12-18 years(boys and girls) with α-thalassemia**

| **Parameter** | **Normal** | | **Silent carrier** | | **Mild** | | **Hb H Disease** | | ***P1*-value** | ***P2*-value** | **References** |
| --- | --- | --- | --- | --- | --- | --- | --- | --- | --- | --- | --- |
|  | **Boys(n=10)** | **Girls(n=9)** | **Boys(n=4)** | **Girls(n=2)** | **Boys(n=0)** | **Girls(n=3)** | **Boys(n=1)** | **Girls(n=9)** | **Boys** | **Girls** |  |
| Hemolysis |  |  |  |  |  |  |  |  |  |  |  |
| RBC(1012/L) | 5.12±0.36 | 4.56±0.18 | 5.15±0.21 | 5.05±0.21 | - | 5.47±0.31 | 6.0 | 4.7±0.74 | 0.074 | 0.078 | 4.1~5.3 |
| HGB (g/L) | 146.5±9.45 | 130±7.7 | 129.45±10.31 | 134±0 | - | 122.27±6.77 | 99 | 96.67±10.89**^abc^** | **<0.001** | **<0.001** | 114~154 |
| HCT (%) | 44.95±2.91 | 40.08±2.16 | 40.9±3.15 | 41±0.57 | - | 39.27±2.35 | 31.8 | 32.61±4.72 **^abc^** | 0.003 | **<0.001** | 36~47 |
| MCV (fL) | 87.89±3.79 | 87.83±4.41 | 79.45±6.99 | 81.75±1.77 | - | 72.43±5.6 | 53.3 | 70.09±11.5**^a^** | **<0.001** | **0.001** | 80~100 |
| MCH (pg) | 28.63±1.38 | 28.46±1.39 | 25.2±2.39 | 26.6±0.99 | - | 22.57±1.68 | 16.7 | 20.86±3.46 **^a^** | **<0.001** | **<0.001** | 25~34 |
| MCHC (g/L) | 325.8±4.96 | 324.11±6.43 | 316.83±6.22 | 325.5±4.95 | - | 311.1±1.15 | 313.0 | 298.78±25.6 **^a^** | **0.017** | **0.03** | 320~360 |
| TBIL(μmol/L) | 12.13±5.75 | 9.3±2.83 | 8.4±4.59 | 9.25±6.01 | - | 11.97±2.57 | 17.5 | 43.09±25.01 **^abc^** | 0.307 | **0.002** | ≤21.0 |
| DBIL(μmol/L) | 3.9±1.77 | 2.79±0.67 | 2.48±1.41 | 2.55±1.91 | - | 4.1±0.7 | 5.9 | 10.4±5.37 **^abc^** | 0.183 | **0.001** | 0.4~6.8 |
| IBIL (μmol/L) | 8.21±4.09 | 6.51±2.34 | 5.88±3.15 | 6.7±4.1 | - | 7.83±1.97 | 11.6 | 32.69±22.14**^a^** | 0.386 | **0.005** | 1.7~17 |
| LDH (U/L) | 193.65±29.34 | 172±23.87 | 228.38±21.69 | 166.5±27.58 | - | 210.83±31.95 | 119.0 | 295.61±168.93 | 0.146 | 0.136 | 120~250 |
| Leukocytes |  |  |  |  |  |  |  |  |  |  |  |
| WBC (109/L) | 7.07±0.95 | 7.33±1.53 | 7.08±1.18 | 6.7±0.57 | - | 5.3±1.68 | 8.9 | 6.81±2.05 | 0.257 | 0.409 | 4.1~11.0 |
| NE# (109/L) | 3.41±0.7 | 3.73±1.14 | 3.8±1.53 | 3.2±0.14 | - | 2.7±0.89 | 3.9 | 3.88±1.16 | 0.747 | 0.415 | 1.8~8.3 |
| LYM#(109/L) | 2.81±0.53 | 2.91±0.53 | 2.68±0.29 | 2.85±0.64 | - | 2.1±0.7 | 2.9 | 2.33±1.01 | 0.865 | 0.313 | 1.2~3.8 |
| MON#(109/L) | 0.47±0.13 | 0.42±0.14 | 0.35±0.06 | 0.35±0.07 | - | 0.3±0.1 | 0.7 | 0.39±0.16 | 0.055 | 0.625 | 0.14~0.74 |
| EO#(109/L) | 0.33±0.17 | 0.22±0.15 | 0.28±0.17 | 0.3±0.28 | - | 0.2±0.1 | 1.3 | 0.19±0.18 | **<0.001** | 0.852 | 0~0.68 |
| Platelets |  |  |  |  |  |  |  |  |  |  |  |
| PLT (109/L) | 278.6±49.49 | 289.44±68.2 | 338.13±89.76 | 300.5±41.72 | - | 285.17±53.76 | 271 | 240.11±101.43 | 0.287 | 0.565 | 150~407 |
| Lipid profile |  |  |  |  |  |  |  |  |  |  |  |
| CHOL(mmol/L) | 4.43±1.32 | 3.97±0.79 | 3.85±1.05 | 4.55±0.35 | - | 4.13±0.15 | 3.2 | 3.03±0.92 | 0.545 | **0.035** | <5.18 |
| TG (mmol/L) | 1.52±0.91 | 1±0.29 | 1.28±0.45 | 0.9±0.14 | - | 0.7±0.26 | 0.5 | 0.8±0.27 | 0.492 | 0.314 | <1.70 |
| HDL (mmol/L) | 1.37±0.24 | 1.52±0.14 | 1.35±0.5 | 1.95±0.35 | - | 1.67±0.25 | 1.4 | 1.07±0.27 **^abc^** | 0.989 | **<0.001** | 1.0~1.6 |
| LDL (mmol/L) | 2.74±1.02 | 2.44±0.67 | 2.53±0.5 | 2.1±0 | - | 2.27±0.4 | 1.6 | 1.83±0.68 | 0.503 | 0.263 | ≤3.3 |
| Myocardial enzyme | |  |  |  |  |  |  |  |  |  |  |
| CK (U/L) | 157.06±71.72 | 80±22.68 | 77.65±23.67 | 69.5±24.75 | - | 92.53±13.46 | 159.0 | 52.56±49.24 | 0.140 | 0.292 | 40~200 |
| CK-MB (U/L) | 10.61±5.13 | 9.79±4.99 | 16.03±3.52 | 12.15±6.72 | - | 16.87±3.49 | 15.7 | 10.7±3.41 | 0.166 | 0.138 | <25 |
| Ferritin |  |  |  |  |  |  |  |  |  |  |  |
| SerumFerritin  (ng/ml) | 77.34±61.67 | 43.29±22.48 | 65.65±57.03 | 79.85±5.44 | - | 81.4±61.28 | 42.8 | 226.57±186.1**^a^** | 0.840 | **0.034** | 11.0~306.8 |
| Liver functions | |  |  |  |  |  |  |  |  |  |  |
| ALT (U/L) | 27±36.17 | 13.78±9.51 | 18.25±7.41 | 14.5±7.78 | - | 11.67±6.03 | 18.0 | 18.22±13.69 | 0.880 | 0.774 | 6~29 |
| AST (U/L) | 24.1±10.79 | 18±4.82 | 24.58±4.42 | 16.5±2.12 | - | 22.43±5.17 | 29.0 | 27.33±18.59 | 0.889 | 0427 | 12~37 |
| ALBP (g/L) | 43.65±1.95 | 43.61±3.39 | 43.05±1.92 | 41.45±1.06 | - | 44.9±1.54 | 43.2 | 43.44±4.56 | 0.866 | 0.793 | 42~56 |
| Renal functions | |  |  |  |  |  |  |  |  |  |  |
| BUN (mmol/L) | 4.91±1.04 | 4.31±1.19 | 6.13±1.19 | 5.45±0.78 | - | 4.53±1.56 | 4.7 | 8.29±11.93 | 0.186 | 0.728 | 2.5~6.5 |
| CREA (μmol/L) | 60.4±12.14 | 44.31±8.99 | 50.03±10.41 | 45.6±8.63 | - | 46.17±3.85 | 38.4 | 42.4±16.4 | 0.138 | 0.963 | 33~75 |
| Coagulation Function | |  |  |  |  |  |  |  |  |  |  |
| PT(s) | 11.64±0.69 | 11.8±0.5 | 11.73±0.35 | 11.5±0.57 | - | 12.03±0.23 | 11.9 | 12.8±1.18 | 0.911 | 0.077 | 9.8~13.2 |
| APTT(s) | 29.98±2.59 | 30.5±3.02 | 32.05±3.99 | 30.6±2.12 | - | 32±1.45 | 32.1 | 34.78±4.57 | 0.471 | 0.110 | 22.5~34.0 |
| Fbg(g/L) | 2.86±0.49 | 3.13±0.8 | 3±0.62 | 2.95±0.21 | - | 2.77±0.35 | 2.8 | 2.8±0.64 | 0.889 | 0.726 | 2.08~3.85 |
| PT-INR | 1.01±0.06 | 1.02±0.04 | 1.0±0 | 1±0 | - | 1.03±0.06 | 1.0 | 1.1±0.09 | 0.934 | 0.074 | 0.85~1.2 |

Notes: Data are presented as mean ± standard deviation (SD);P1-value stands for Boys differences among the four groups, P2-value stands for Girls differences among the four groups; Bold Signifies *P*<0.05;**^a^** Compared with normal group, *P* <0.05; **^b^** Compared with the Silent carrier group, *P* <0.05; **^c^** Compared with mild group, *P* <0.05;

Abbreviations: RBC, red blood cell; HGB, hemoglobin:; HCT, hematocrit; MCV, mean corpuscular volume; MCH, mean hemoglobin concentration; MCHC, mean corpuscular hemoglobin concentration; TBIL, total bilirubin; DBIL, direct bilirubin; IBIL, indirect bilirubin; LDH, lactate dehydrogenase; WBC, white blood cell; NE#, neutrophil count; LYM#, lymphocyte count; MON#, monocyte count; EO#, eosinophil count; PLT, platelet;CHOL, cholesterol; TG, triglyceride; HDL, high density lipoprotein; LDL, low density lipoprotein; CK, creatine kinase; CK-MB, Creatine Kinase Isoenzyme-MB; SerumFerritin;ALT, alanine aminotransferase; AST, aspartate aminotransferase; ALBP, alpha-1-acid glycoprotein; BUN, blood urea nitrogen; CREA, creatinine;PT, prothrombin time; APTT, activated partial thromboplastin time; Fbg, fibrinogen; PT-INR, prothrombin time - international normalized ratio.
