# Supplementary material for: Analysis of clinical parameters of different types of α-thalassemia children in Hainan region, China
Source: PeerJ. 2026 Jan 8;14:e20586. doi: 10.7717/peerj.20586 (PMC12790785; doi:10.7717/peerj.20586)
Supplement: Supplemental Information 5 [file peerj-14-20586-s005.docx]

**Supplementary table 4. Hb H Disease Blood characteristics of children**

| **Parameter**  **Total** | **Deletional**  **N=80** | **Non-deletional**  **N=18** | **P-value** | **References** |
| --- | --- | --- | --- | --- |
| RBC(10^12^/L) | 5.65±0.83 | 5.21±0.92 | **0.049** | 4.1~5.3 |
| HGB (g/L) | 96.58±8.9 | 105.06±15.6 | **0.002** | 114~154 |
| HCT (%) | 31.53±3.04 | 34.86±4.18 | **<0.001** | 36~47 |
| MCV (fL) | 55.5±5.94 | 67.99±8.33 | **<0.001** | 80~100 |
| MCH (pg) | 17.04±1.81 | 20.39±2.36 | **<0.001** | 25~34 |
| MCHC (g/L) | 307.15±9.47 | 301.33±25.39 | 0.107 | 320~360 |
| WBC (10^9^/L) | 7.55±2.09 | 6.77±1.71 | 0.144 | 4.1~11.0 |
| NE# (10^9^/L) | 3.88±1.72 | 3.39±0.95 | 0.249 | 1.8~8.3 |
| LYM#(10^9^/L) | 2.82±0.75 | 2.72±0.99 | 0.615 | 1.2~3.8 |
| MON#(10^9^/L) | 0.56±0.7 | 0.4±0.14 | 0.333 | 0.14~0.74 |
| EO#(10^9^/L) | 0.41±0.58 | 0.27±0.21 | 0.293 | 0~0.68 |
| PLT (10^9^/L) | 404.64±127.15 | 307.06±114.87 | **0.004** | 150~407 |
| **Parameter**  **1-5 years** | **Deletional**  **N=25** | **Non-deletional**  **N=4** | **P-value** | **References** |
| RBC(10^12^/L) | 5.71±0.55 | 5.18±0.88 | 0.107 | 4.1~5.3 |
| HGB (g/L) | 96.19±8.26 | 93±20.7 | 0.574 | 114~154 |
| HCT (%) | 31.22±2.78 | 31.65±4.49 | 0.792 | 36~47 |
| MCV (fL) | 55.2±6.77 | 61.6±3.78 | 0.079 | 80~100 |
| MCH (pg) | 17.03±2.19 | 17.98±1.21 | 0.411 | 25~34 |
| MCHC (g/L) | 308.49±9.76 | 292.75±30.04 | **0.041** | 320~360 |
| WBC (10^9^/L) | 8.56±2.53 | 6.93±1.88 | 0.229 | 4.1~11.0 |
| NE# (10^9^/L) | 4.58±2.21 | 3.15±0.88 | 0.218 | 1.8~8.3 |
| LYM#(10^9^/L) | 3.05±1.05 | 3.2±1.12 | 0.797 | 1.2~3.8 |
| MON#(10^9^/L) | 0.81±1.21 | 0.35±0.1 | 0.459 | 0.14~0.74 |
| EO#(10^9^/L) | 0.34±0.3 | 0.25±0.13 | 0.593 | 0~0.68 |
| PLT (10^9^/L) | 437.54±139.66 | 388.75±97.33 | 0.510 | 150~407 |
| **Parameter**  **6-11 years** | **Deletional**  **N=51** | **Non-deletional**  **N=8** | **P-value** | **References** |
| RBC(10^12^/L) | 5.63±0.95 | 5.79±0.68 | 0.658 | 4.1~5.3 |
| HGB (g/L) | 97.18±9.12 | 114.38±12.19 | **<0.001** | 114~154 |
| HCT (%) | 31.81±3.13 | 36.88±3.01 | **<0.001** | 36~47 |
| MCV (fL) | 55.66±5.78 | 64.33±5.45 | **<0.001** | 80~100 |
| MCH (pg) | 17.05±1.7 | 19.81±0.49 | **<0.001** | 25~34 |
| MCHC (g/L) | 306.59±9.65 | 309.38±17.61 | 0.505 | 320~360 |
| WBC (10^9^/L) | 7.05±1.72 | 6.85±1.1 | 0.758 | 4.1~11.0 |
| NE# (10^9^/L) | 3.51±1.38 | 3.28±0.72 | 0.641 | 1.8~8.3 |
| LYM#(10^9^/L) | 2.73±0.54 | 2.8±0.79 | 0.747 | 1.2~3.8 |
| MON#(10^9^/L) | 0.44±0.13 | 0.46±0.13 | 0.677 | 0.14~0.74 |
| EO#(10^9^/L) | 0.45±0.68 | 0.33±0.24 | 0.626 | 0~0.68 |
| PLT (10^9^/L) | 394.87±121.78 | 354.25±73.47 | 0.365 | 150~407 |
| **Parameter**  **12-18 years** | **Deletional**  **N=4** | **Non-deletional**  **N=6** | **P-value** | **References** |
| RBC(10^12^/L) | 5.4±0.59 | 4.45±0.74 | 0.065 | 4.1~5.3 |
| HGB (g/L) | 91.25±10.34 | 100.67±9.16 | 0.168 | 114~154 |
| HCT (%) | 29.88±3.42 | 34.3±4.4 | 0.130 | 36~47 |
| MCV (fL) | 55.3±1.94 | 77.15±5.55 | **<0.001** | 80~100 |
| MCH (pg) | 16.93±0.36 | 22.78±2.38 | **0.001** | 25~34 |
| MCHC (g/L) | 306±5.23 | 296.33±31.99 | 0.573 | 320~360 |
| WBC (10^9^/L) | 7.7±1.15 | 6.57±2.47 | 0.422 | 4.1~11.0 |
| NE# (10^9^/L) | 4.15±0.81 | 3.7±1.29 | 0.555 | 1.8~8.3 |
| LYM#(10^9^/L) | 2.53±0.78 | 2.3±1.15 | 0.742 | 1.2~3.8 |
| MON#(10^9^/L) | 0.53±0.17 | 0.35±0.16 | 0.143 | 0.14~0.74 |
| EO#(10^9^/L) | 0.45±0.57 | 0.2±0.21 | 0.348 | 0~0.68 |
| PLT (10^9^/L) | 323.5±55.56 | 189.67±78.63 | **0.019** | 150~407 |

Notes: Data are presented as mean ± standard deviation (SD);P-value stands for differences among the four groups; Bold Signifies P<0.05;

Abbreviations: N, number; RBC, red blood cell; HGB, hemoglobin:; HCT, hematocrit; MCV, mean corpuscular volume; MCH, mean hemoglobin concentration; MCHC, mean corpuscular hemoglobin concentration; WBC, white blood cell; NE#, neutrophil count; LYM#, lymphocyte count; MON#, monocyte count; EO#, eosinophil count; PLT, platelet.
