# Supplementary material for: Analysis of clinical parameters of different types of α-thalassemia children in Hainan region, China
Source: PeerJ. 2026 Jan 8;14:e20586. doi: 10.7717/peerj.20586 (PMC12790785; doi:10.7717/peerj.20586)
Supplement: Supplemental Information 6 [file peerj-14-20586-s006.docx]

**Supplementary table 5. Biochemical and ferritin characteristics of children with deletional VS. non-deletional Hb H disease**

| **Parameter**  **Total** | **Deletional**  **N=80** | **Non-deletional**  **N=18** | **P-value** | **References** |
| --- | --- | --- | --- | --- |
| **Bilirubin metabolism and LDH** | |  |  |  |
| TBIL(μmol/L) | 15.67±12.36 | 30.52±33.53 | **0.002** | ≤21.0 |
| DBIL(μmol/L) | 4.56±1.96 | 6.38±5.57 | **0.020** | 0.4~6.8 |
| IBIL (μmol/L) | 9.94±5.6 | 24.14±30.03 | **<0.001** | 1.7~17 |
| **Lipid profile** |  |  |  |  |
| CHOL(mmol/L) | 3.62±0.84 | 3.47±0.97 | 0.523 | <5.18 |
| TG (mmol/L) | 0.81±0.34 | 0.77±0.28 | 0.633 | <1.70 |
| HDL (mmol/L) | 1.3±0.23 | 1.36±0.43 | 0.461 | 1.0~1.6 |
| LDL (mmol/L) | 2.15±0.74 | 2.02±0.66 | 0.491 | ≤3.3 |
| **Myocardial enzyme** | |  |  |  |
| CK (U/L) | 93.38±35.71 | 113.61±118.36 | 0.195 | 40~200 |
| CK-MB (U/L) | 20.1±8.09 | 18.49±7.49 | 0.442 | <25 |
| LDH (U/L) | 246.7±45.26 | 308.58±123.01 | **<0.001** | 120~250 |
| **Liver functions** |  |  |  |  |
| ALT (U/L) | 14.11±5.27 | 17.72±9.86 | **0.031** | 6~29 |
| AST (U/L) | 32.38±29.77 | 31.17±12.5 | 0.867 | 12~37 |
| ALBP (g/L) | 43.73±2.36 | 43.49±3.59 | 0.724 | 42~56 |
| **Renal functions** | |  |  |  |
| BUN (mmol/L) | 4.55±1.11 | 6.46±8.43 | **0.050** | 2.5~6.5 |
| CREA (μmol/L) | 33.75±8.59 | 32.66±12.44 | 0.657 | 33~75 |
| **Serum Ferritin** (ng/ml) | 185.92±211.03 | 165.29±160.87 | 0.698 | 11.0~306.8 |
| **Parameter**  **1-5 years** | **Deletional**  **N=25** | **Non-deletional**  **N=4** | **P-value** | **References** |
| **Bilirubin metabolism and LDH** | |  |  |  |
| TBIL(μmol/L) | 18.24±19.71 | 16.45±10.35 | 0.862 | ≤21.0 |
| DBIL(μmol/L) | 4.25±1.73 | 5.3±3.49 | 0.340 | 0.4~6.8 |
| IBIL (μmol/L) | 10.28±6.81 | 11.18±6.95 | 0.809 | 1.7~17 |
| **Lipid profile** |  |  |  |  |
| CHOL(mmol/L) | 3.78±0.78 | 3.6±0.59 | 0.663 | <5.18 |
| TG (mmol/L) | 0.8±0.45 | 0.88±0.25 | 0.738 | <1.70 |
| HDL (mmol/L) | 1.28±0.21 | 1.3±0.16 | 0.830 | 1.0~1.6 |
| LDL (mmol/L) | 2.32±0.69 | 2.2±0.62 | 0.754 | ≤3.3 |
| **Myocardial enzyme** | |  |  |  |
| CK (U/L) | 97.58±33.17 | 82±9.13 | 0.365 | 40~200 |
| CK-MB (U/L) | 22.54±9.6 | 23.15±7.12 | 0.904 | <25 |
| LDH (U/L) | 269.5±43.54 | 299.5±42.6 | 0.211 | 120~250 |
| **Liver functions** |  |  |  |  |
| ALT (U/L) | 14.28±6.14 | 14.5±4.73 | 0.964 | 6~29 |
| AST (U/L) | 32.01±7.21 | 32±3.74 | 0.997 | 12~37 |
| ALBP (g/L) | 43.62±1.92 | 45.25±3.08 | 0.158 | 42~56 |
| **Renal functions** | |  |  |  |
| BUN (mmol/L) | 4.84±0.86 | 3.78±0.69 | **0.026** | 2.5~6.5 |
| CREA (μmol/L) | 29.09±8.33 | 27.95±7.66 | 0.799 | 33~75 |
| **Serum Ferritin**  (ng/ml) | 135.86±115.41 | 165.8±141.22 | 0.643 | 11.0~306.8 |
| **Parameter**  **6-11 years** | **Deletional**  **N=51** | **Non-deletional**  **N=8** | **P-value** | **References** |
| **Bilirubin metabolism and LDH** | |  |  |  |
| TBIL(μmol/L) | 13.78±6.18 | 21.66±39.97 | 0.177 | ≤21.0 |
| DBIL(μmol/L) | 4.48±1.9 | 3.04±2.32 | 0.058 | 0.4~6.8 |
| IBIL (μmol/L) | 9.27±4.54 | 18.61±37.69 | 0.082 | 1.7~17 |
| **Lipid profile** |  |  |  |  |
| CHOL(mmol/L) | 3.57±0.87 | 3.79±1.02 | 0.524 | <5.18 |
| TG (mmol/L) | 0.82±0.28 | 0.66±0.27 | 0.128 | <1.70 |
| HDL (mmol/L) | 1.33±0.23 | 1.59±0.48 | **0.017** | 1.0~1.6 |
| LDL (mmol/L) | 2.09±0.76 | 2.13±0.69 | 0.914 | ≤3.3 |
| **Myocardial enzyme** | |  |  |  |
| CK (U/L) | 93.17±34.75 | 170.5±156.74 | **0.002** | 40~200 |
| CK-MB (U/L) | 19.56±7 | 21.94±5.64 | 0.365 | <25 |
| LDH (U/L) | 239.46±41.11 | 285.38±93.84 | **0.020** | 120~250 |
| **Liver functions** |  |  |  |  |
| ALT (U/L) | 14.31±4.79 | 15.13±6.33 | 0.672 | 6~29 |
| AST (U/L) | 33.47±36.9 | 30.13±5.74 | 0.801 | 12~37 |
| ALBP (g/L) | 43.76±2.39 | 42.96±3.29 | 0.408 | 42~56 |
| **Renal functions** | |  |  |  |
| BUN (mmol/L) | 4.46±1.21 | 4.59±1.21 | 0.790 | 2.5~6.5 |
| CREA (μmol/L) | 34.85±6.79 | 31.36±9.98 | 0.211 | 33~75 |
| **Serum Ferritin**  (ng/ml) | 218.47±246.24 | 70.63±50.14 | 0.098 | 11.0~306.8 |
| **Parameter**  **12-18 years** | **Deletional**  **N=4** | **Non-deletional**  **N=6** | **P-value** | **References** |
| **Bilirubin metabolism and LDH** | |  |  |  |
| TBIL(μmol/L) | 23.75±8.67 | 51.72±26.43 | 0.079 | ≤21.0 |
| DBIL(μmol/L) | 7.55±1.95 | 11.55±6.31 | 0.262 | 0.4~6.8 |
| IBIL (μmol/L) | 16.2±7.16 | 40.17±23.61 | 0.089 | 1.7~17 |
| **Lipid profile** |  |  |  |  |
| CHOL(mmol/L) | 3.18±0.68 | 2.97±1.02 | 0.732 | <5.18 |
| TG (mmol/L) | 0.68±0.22 | 0.83±0.31 | 0.404 | <1.70 |
| HDL (mmol/L) | 1.13±0.22 | 1.08±0.33 | 0.832 | 1.0~1.6 |
| LDL (mmol/L) | 1.88±0.69 | 1.77±0.68 | 0.812 | ≤3.3 |
| **Myocardial enzyme** | |  |  |  |
| CK (U/L) | 69.75±61.31 | 58.83±60.05 | 0.787 | 40~200 |
| CK-MB (U/L) | 11.8±4.85 | 10.8±2.92 | 0.691 | <25 |
| LDH (U/L) | 196.5±45.05 | 345.58±188.29 | 0.166 | 120~250 |
| **Liver functions** |  |  |  |  |
| ALT (U/L) | 10.5±5.26 | 23.33±14.29 | 0.129 | 6~29 |
| AST (U/L) | 20.75±5.68 | 32±21.75 | 0.350 | 12~37 |
| ALBP (g/L) | 44.03±4.64 | 43.02±4.46 | 0.739 | 42~56 |
| **Renal functions** | |  |  |  |
| BUN (mmol/L) | 3.73±0.69 | 10.73±14.35 | 0.367 | 2.5~6.5 |
| CREA (μmol/L) | 48.73±10.82 | 37.52±17.4 | 0.288 | 33~75 |
| **Serum Ferritin**  (ng/ml) | 83.73±53.02 | 291.17±197.86 | 0.079 | 11.0~306.8 |

Notes: Data are presented as mean ± standard deviation (SD);P-value stands for differences among the four groups; Bold Signifies P<0.05;

Abbreviations: N, number; TBIL, total bilirubin; DBIL, direct bilirubin; IBIL, indirect bilirubin; LDH, lactate dehydrogenase; CHOL, cholesterol; TG, triglyceride; HDL, high density lipoprotein; LDL, low density lipoprotein; CK, creatine kinase; CK-MB, Creatine Kinase Isoenzyme-MB; Serum Ferritin; ALT, alanine aminotransferase; AST, aspartate aminotransferase; ALBP, alpha-1-acid glycoprotein; BUN, blood urea nitrogen; CREA, creatinine.
