# Supplementary material for: Analysis of clinical parameters of different types of α-thalassemia children in Hainan region, China
Source: PeerJ. 2026 Jan 8;14:e20586. doi: 10.7717/peerj.20586 (PMC12790785; doi:10.7717/peerj.20586)
Supplement: Supplemental Information 7 [file peerj-14-20586-s007.docx]

**Supplementary table 6. Coagulation function characteristics of children with deletional VS. non-deletional Hb H disease**

| **Parameter**  **Total** | **Deletional**  **N=80** | **Non-deletional**  **N=18** | **P-value** | **References** |
| --- | --- | --- | --- | --- |
| PT(s) | 12.17±0.82 | 12.28±0.99 | 0.639 | 9.8~13.2 |
| APTT(s) | 33.57±7.93 | 33.75±3.09 | 0.923 | 22.5~34.0 |
| Fbg(g/L) | 2.7±0.55 | 2.81±0.52 | 0.433 | 2.08~3.85 |
| PT-INR | 1.04±0.08 | 1.06±0.08 | 0.521 | 0.85~1.2 |
| **Parameter**  **1-5 years** | **Deletional**  **N=25** | **Non-deletional**  **N=4** | **P-value** | **References** |
| PT(s) | 12.21±0.75 | 11.55±0.37 | 0.100 | 9.8~13.2 |
| APTT(s) | 34.6±12.45 | 32.78±1.64 | 0.775 | 22.5~34.0 |
| Fbg(g/L) | 2.88±0.71 | 2.6±0.29 | 0.439 | 2.08~3.85 |
| PT-INR | 1.05±0.07 | 1±0 | 0.197 | 0.85~1.2 |
| **Parameter**  **6-11 years** | **Deletional**  **N=51** | **Non-deletional**  **N=8** | **P-value** | **References** |
| PT(s) | 12.11±0.84 | 12.34±0.82 | 0.477 | 9.8~13.2 |
| APTT(s) | 33.06±4.75 | 33.15±2.53 | 0.960 | 22.5~34.0 |
| Fbg(g/L) | 2.58±0.41 | 3.06±0.55 | 0.005 | 2.08~3.85 |
| PT-INR | 1.04±0.08 | 1.05±0.08 | 0.674 | 0.85~1.2 |
| **Parameter**  **12-18 years** | **Deletional**  **N=4** | **Non-deletional**  **N=6** | **P-value** | **References** |
| PT(s) | 12.75±1.06 | 12.68±1.31 | 0.935 | 9.8~13.2 |
| APTT(s) | 33.48±5.03 | 35.2±4.24 | 0.574 | 22.5~34.0 |
| Fbg(g/L) | 3.08±0.69 | 2.62±0.51 | 0.259 | 2.08~3.85 |
| PT-INR | 1.08±0.1 | 1.1±0.09 | 0.684 | 0.85~1.2 |

Notes: Data are presented as mean ± standard deviation (SD);P-value stands for differences among the four groups; Bold Signifies P<0.05;

Abbreviations: N, number; PT, prothrombin time; APTT, activated partial thromboplastin time; Fbg, fibrinogen; PT-INR, prothrombin time - international normalized ratio.
