# Supplementary material for: Analysis of clinical parameters of different types of α-thalassemia children in Hainan region, China
Source: PeerJ. 2026 Jan 8;14:e20586. doi: 10.7717/peerj.20586 (PMC12790785; doi:10.7717/peerj.20586)
Supplement: Supplemental Information 8 [file peerj-14-20586-s008.docx]

**Supplementary table 7. Hematologic and biochemical characteristics among deletional and non-deletional Hb H Disease difference genotypes**

| **Parameter**  **Total** | | **Deletional Hb H disease** | | **Non-Deletional Hb H disease** | | |  |  |  |
| --- | --- | --- | --- | --- | --- | --- | --- | --- | --- |
|  |  | **--^SEA^/-α^3.7^**  **n=39** | **--^SEA^/-α^4.2^**  **n=41** | **--^SEA^/α^WS^α**  **n=10** | **--^SEA^/α^QS^α**  **n=3** | **--^SEA^/α^CS^ α**  **n=5** | ***P*-value** | **References** |  |
| **Blood cells** | |  |  |  |  |  |  |  |  |
| RBC(10^12^/L) | | 5.61±1.06 | 5.68±0.53 | 5.92±0.43 | 4.7±0.35 | 4.08±0.29**^abc^** | **<0.001** | 4.1~5.3 |  |
| HGB (g/L) | | 96.69±9.25 | 96.47±8.67 | 116±9.13**^ab^** | 84±13.45**^c^** | 95.8±4.15**^c^** | **<0.001** | 114~154 |  |
| HCT (%) | | 31.68±3.34 | 31.39±2.75 | 36.87±3.25**^ab^** | 32.63±6.25 | 32.16±2.8 | **<0.001** | 36~47 |  |
| MCV (fL) | | 55.32±6.7 | 55.66±5.19 | 62.37±3.24 **^ab^** | 69.5±9.25**^ab^** | 78.34±3.69**^abc^** | **<0.001** | 80~100 |  |
| MCH (pg) | | 16.98±2.05 | 17.09±1.58 | 19.61±0.64 **^ab^** | 17.97±1.66 | 23.42±2.0**^abcd^** | **<0.001** | 25~34 |  |
| MCHC (g/L) | | 307.16±10.46 | 307.14±8.56 | 314.8±6.96 | 259.33±14.19**^abc^** | 299.6±27.37**^d^** | **<0.001** | 320~360 |  |
| WBC (10^9^/L) | | 7.66±2.41 | 7.44±1.76 | 7.2±1.18 | 5.6±0.79 | 6.62±2.73 | 0.435 | 4.1~11.0 |  |
| NE# (10^9^/L) | | 4.04±2.08 | 3.72±1.31 | 3.52±0.77 | 3.03±1.07 | 3.34±1.32 | 0.689 | 1.8~8.3 |  |
| LYM#(10^9^/L) | | 2.77±0.88 | 2.86±0.62 | 2.95±0.87 | 2.17±1.25 | 2.6±1.14 | 0.588 | 1.2~3.8 |  |
| MON#(10^9^/L) | | 0.48±0.21 | 0.63±0.95 | 0.43±0.14 | 0.3±0.1 | 0.4±0.16 | 0.716 | 0.14~0.74 |  |
| EO#(10^9^/L) | | 0.38±0.39 | 0.45±0.72 | 0.31±0.22 | 0.13±0.06 | 0.26±0.22 | 0.801 | 0~0.68 |  |
| PLT (10^9^/L) | | 395.04±146.26 | 413.77±106.92 | 324.9±110.55 | 379.67±153.03 | 227.8±65.32**^ab^** | **0.017** | 150~407 |  |
| **Bilirubin metabolism** | | |  |  |  |  |  |  |  |
| TBIL(μmol/L) | | 13.11±6.69 | 18.1±15.7 | 7.65±2.36 | 36.53±21.34**^ac^** | 72.66±31.35**^abcd^** | **<0.001** | ≤21.0 |  |
| DBIL(μmol/L) | | 4.12±1.8 | 4.99±2.03 | 2.26±0.6**^b^** | 10.83±4.74**^abc^** | 11.94±4.89**^abc^** | **<0.001** | 0.4~6.8 |  |
| IBIL (μmol/L) | | 9±5.27 | 10.82±5.83 | 5.37±1.98 | 25.73±16.57**^ac^** | 60.74±33.61**^abcd^** | **<0.001** | 1.7~17 |  |
| **Lipid profile** | |  |  |  |  |  |  |  |  |
| CHOL(mmol/L) | | 3.76±0.99 | 3.48±0.64 | 3.99±0.61 | 3.17±1.2 | 2.62±0.9**^ac^** | **0.017** | <5.18 |  |
| TG (mmol/L) | | 0.85±0.37 | 0.77±0.3 | 0.66±0.24 | 1.07±0.15 | 0.8±0.31 | 0.291 | <1.70 |  |
| HDL (mmol/L) | | 1.33±0.23 | 1.28±0.23 | 1.62±0.34**^ab^** | 1.13±0.29**^c^** | 0.96±0.26**^ac^** | **<0.001** | 1.0~1.6 |  |
| LDL (mmol/L) | | 2.28±0.88 | 2.03±0.55 | 2.27±0.5 | 1.97±1.03 | 1.56±0.59 | 0.199 | ≤3.3 |  |
| **Myocardial enzyme** | | |  |  |  |  |  |  |  |
| CK (U/L) | | 98.16±34.35 | 88.83±36.8 | 167.5±137.26**^ab^** | 58.33±28.11 **^c^** | 39±15.41**^c^** | **<0.001** | 40~200 |  |
| CK-MB (U/L) | | 19.89±7.36 | 20.3±8.82 | 23.67±5.19 | 15.33±4.73 | 10.04±1.76**^abc^** | **0.024** | <25 |  |
| LDH (U/L) | | 251.74±50.54 | 241.9±39.63 | 257.6±35.15 | 428.67±196.36**^abc^** | 338.5±154.31**^ab^** | **<0.001** | 120~250 |  |
| **Serum Ferritin**  (ng/ml) | 188.83±205.97 | | 183.15±218.26 | 54.77±24.35 | 382.4±164.66 | 256.06±137.49 | 0.103 | 11.0~306.8 |  |
| **Liver functions** | |  |  |  |  |  |  |  |  |
| ALT (U/L) | | 14.82±6.04 | 13.44±4.37 | 15.4±6.31 | 14.67±5.51 | 24.2±15.35 | **0.011** | 6~29 |  |
| AST (U/L) | | 30.15±7.59 | 34.49±41.07 | 28.6±6.0 | 31.67±4.73 | 36±22.92 | 0.944 | 12~37 |  |
| ALBP (g/L) | | 43.74±2.18 | 43.72±2.54 | 43.36±3.32 | 47.13±3.88 | 41.56±2.83 | 0.064 | 42~56 |  |
| **Renal functions** | |  |  |  |  |  |  |  |  |
| BUN (mmol/L) | | 4.73±1.06 | 4.37±1.15 | 4.41±1.19 | 4.43±1.04 | 11.76±15.8**^abcd^** | **<0.001** | 2.5~6.5 |  |
| CREA (μmol/L) | | 33.04±7.98 | 34.41±9.18 | 33.14±9.98 | 30.33±6.66 | 33.08±20.16 | 0.935 | 33~75 |  |
| **Coagulation function** | | |  |  |  |  |  |  |  |
| PT(s) | | 12.04±0.73 | 12.3±0.89 | 12.08±0.9 | 11.8±0.4 | 12.96±1.19 | 0.155 | 9.8~13.2 |  |
| APTT(s) | | 32.75±3.21 | 34.34±10.63 | 32.78±2.36 | 33.97±1.57 | 35.56±4.5 | 0.840 | 22.5~34.0 |  |
| Fbg(g/L) | | 2.72±0.56 | 2.68±0.55 | 3.04±0.49 | 2.23±0.21 | 2.7±0.44 | 0.196 | 2.08~3.85 |  |
| PT-INR | | 1.03±0.07 | 1.05±0.08 | 1.04±0.07 | 1.03±0.06 | 1.1±0.1 | 0.443 | 0.85~1.2 |  |

Notes: Data are presented as mean ± standard deviation (SD);P-value stands for differences among the four groups; Bold Signifies P<0.05;

1. Compared with the --^SEA^/-α^3.7^ group, P <0.05; b, Compared with the **--**^SEA^/-α^4.2^group, P <0.05; c, Compared with the --^SEA^/α^WS^α group, P <0.05;

d, Compared with the --^SEA^/α^QS^α group, P <0.05;

Abbreviations: n, number; RBC, red blood cell; HGB, hemoglobin:; HCT, hematocrit; MCV, mean corpuscular volume; MCH, mean hemoglobin concentration; MCHC, mean corpuscular hemoglobin concentration; WBC, white blood cell; NE#, neutrophil count; LYM# lymphocyte count; MON#, monocyte count; EO#, eosinophil count; PLT, platelet ;TBIL, total bilirubin; DBIL, direct bilirubin; IBIL, indirect bilirubin; LDH, lactate dehydrogenase; CHOL, cholesterol; TG, triglyceride; HDL, high density lipoprotein; LDL, low density lipoprotein; CK, creatine kinase; CK-MB, Creatine Kinase Isoenzyme-MB; Serum Ferritin; ALT, alanine aminotransferase; AST, aspartate aminotransferase; ALBP, alpha-1-acid glycoprotein; BUN, blood urea nitrogen; CREA, creatinine; PT, prothrombin time; APTT, activated partial thromboplastin time; Fbg, fibrinogen; PT-INR, prothrombin time - international normalized ratio.
